# Metabolomic Changes in Rat Serum after Chronic Exposure to Glyphosate-Based Herbicide

**DOI:** 10.3390/metabo14010050

**Published:** 2024-01-13

**Authors:** Oluwatosin Daramola, Cristian D. Gutierrez Reyes, Jesús Chávez-Reyes, Bruno A. Marichal-Cancino, Judith Nwaiwu, Sherifdeen Onigbinde, Moyinoluwa Adeniyi, Joy Solomon, Md Mostofa Al Amin Bhuiyan, Yehia Mechref

**Affiliations:** 1 Department of Chemistry and Biochemistry, Texas Tech University, Lubbock, TX 79409, USA; odaramol@ttu.edu (O.D.); cristian.d.gutierrez-reyes@ttu.edu (C.D.G.R.); jnwaiwu@ttu.edu (J.N.); sonigbin@ttu.edu (S.O.); moadeniy@ttu.edu (M.A.); joy.solomon@ttu.edu (J.S.); mdmobhui@ttu.edu (M.M.A.A.B.); 2 Center of Basic Sciences, Department of Physiology and Pharmacology, Universidad Autónoma de Aguascalientes, Ags, CP 20131, Mexico; jesus.chavezr@edu.uaa.mx (J.C.-R.); bruno.marichal@edu.uaa.mx (B.A.M.-C.)

**Keywords:** glyphosate exposure, rat serum, metabolites, neurological disorders, LC-MS/MS

## Abstract

Glyphosate-based herbicides (GBHs) have gained extensive popularity in recent decades. For many years, glyphosate has been regarded as harmless or minimally toxic to mammals due to the absence of its primary target, the shikimic acid pathway in humans. Nonetheless, mounting evidence suggests that glyphosate may cause adverse health effects in humans via other mechanisms. In this study, we described the metabolomic changes in the serum of experimental rats exposed to chronic GBH using the highly sensitive LC-MS/MS technique. We investigated the possible relationship between chronic exposure to GBH and neurological disorders. Our findings suggest that chronic exposure to GBH can alter spatial learning memory and the expression of some important metabolites that are linked to neurophysiological disorders in young rats, with the female rats showing higher susceptibility compared to the males. This indicates that female rats are more likely to show early symptoms of the disorder on exposure to chronic GBH compared to male rats. We observed that four important metabolites (paraxanthine, epinephrine, L-(+)-arginine, and D-arginine) showed significant changes and involvement in neurological changes as suggested by ingenuity pathway analysis. In conclusion, our results indicate that chronic exposure to GBH can increase the risk of developing neurological disorders.

## 1. Introduction

Glyphosate-based herbicides (GBHs) have gained extensive popularity in recent decades [1,2]. Since its introduction to the market in 1974, glyphosate’s popularity has consistently increased due to its efficacy, affordability, low toxicity, and easy application [2]. Currently, glyphosate utilization in agriculture is estimated to be over 1.6 billion kilograms in the US and 8.6 billion kilograms worldwide [3]. Due to its widespread use as an effective herbicide, glyphosate residues in crops have increased to levels that call for serious attention. Recent years have seen a dramatic increase in glyphosate residues in crops due to their use as drying agents in addition to their traditional use as herbicides for weed management [4].

For many years, glyphosate has been regarded as harmless or minimally toxic to mammals due to the absence of its primary target, the shikimic acid pathway in humans [2]. Nonetheless, mounting evidence suggests that glyphosate may cause adverse health effects in humans via other mechanisms that require thorough investigation [5]. Glyphosate exposure has been implicated in increased breast cancer risk [6] and the stimulation of neuroinflammation that may lead to neurodegenerative disorders [7]. Glyphosate and GBH exposure to GBH have been linked to neurobehavioral changes that stem from the impairment of neuronal developmental processes in mice [8]. Furthermore, glyphosate is reportedly genotoxic, causing DNA damage and chromosomal instability in humans [9].

Omics is an emerging field that holds promise to shine light on the molecular mechanisms causing diseases [10]. Most recently, proteomics, glycomics, glycoproteomics, and metabolomics changes have been implicated in several diseases [11,12,13,14,15,16]. To facilitate better understanding of diseases, many omics fields have advanced methods for better identification of glycans, glycopeptides, and their isomers [17,18,19,20], and have developed several bioinformatic tools [21] that improve interpretations of omics data. With most research focusing on protein changes, metabolomics studies are shedding fresh light on additional types of molecules thought to be important in disease progression [10].

Metabolomics is the comprehensive study of small molecular weight metabolites (<1500 Dalton), their dynamic changes in expression, and interaction in biological systems [22]. Metabolites are highly heterogenous groups of small molecules, including amino acids, lipids, and carbohydrates that are downstream intermediates or products of metabolic pathways [23]. Changes in the concentration of metabolites indicate the current states in complex biological systems, and their assessment can be useful for phenotyping [24]. Because the expression of different metabolites in complex biological systems is influenced by factors such as environment, diet, and disease, metabolomic analysis has gained attention for its ability to identify new biomarkers in different diseases [22,25,26,27,28]. Moreover, the analysis of metabolites might shed light on the pathophysiology of different disease conditions and assess the risk of diseases by identifying unusual biochemical fluxes in metabolic pathways [29].

Comprehensive analysis of metabolites continues to be an analytical challenge because of the variety and wide dynamic range of complex biological systems [30,31]. Extracted metabolites can be quantified using numerous analytical approaches. MS-based approaches provide for the most sensitive detection and, thus, the most comprehensive coverage of the metabolome [32,33]. Liquid chromatography in combination with tandem mass spectrometry (LC-MS/MS) has found wide application in metabolomics analysis because of its high sensitivity, precision, and versatility [34,35]. Coupled with these advantages, LC-MS/MS analysis can be conducted in both positive and negative ionization modes to increase coverage of metabolites with a wide range of polarity [36]. LC-MS-based metabolomic approaches have been employed in the discovery of biomarkers and the clinical evaluation of several chronic disease conditions [37,38,39]. In this study, we described the metabolomic changes in the serum of experimental rats exposed to glyphosate using the highly sensitive LC-MS/MS technique. Serum samples were extracted with DCM/methanol and separated by hydrophilic interaction LC. MS detection was carried out in both positive and negative ionization modes, resulting in significantly enhanced metabolites coverage.

## 2. Materials and Methods

### 2.1. Materials

HPLC-grade methanol (MeOH), water, acetonitrile (ACN), dichloromethane (DCM), and formic acid (FA) were acquired from Fisher Scientific (Fair Lawn, NJ, USA. The GBH used for this research was the Rival^®^ herbicide from Monsanto (St. Louis, MO, USA). Pentobarbital sodium was purchased from PETS Pharma Ltd. ((Mex., Mexico)). Further details on the materials used are provided in Supplementary Table S1.

### 2.2. Animal Study

**Figure 1:**
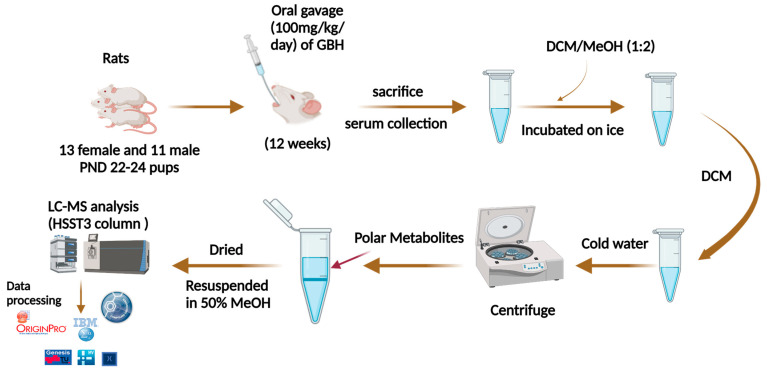
Workflow for the metabolomics analysis using LC-MS method. The data were normalized based on the total abundance of the expression of the metabolites across the samples. Then, parallel reaction monitoring (PRM) analysis was performed to validate the significant metabolites. Workflow created using BioRender.com.

A total of 24 Sprague Dawley (SD) rats; 11 males (6 control and 5 GBH-exposed) and 13 females (6 control and 7 GBH-exposed), at postnatal days 22–24 were obtained from the institutional vivarium of the Autonomous University of Aguascalientes. The methods for the experiments were based on the Mexican Guidelines for Animal Care NOM-062-ZOO-1999 and the National Research Council Guide for the Care and Use of Laboratory Animals [40]. Rats were kept under controlled conditions; 12 h light/dark with light on 7:00 h, temperature of 20–22 °C, humidity of 45–55%, and food and water were available ad libitum. Twelve SD rats (6 males and 6 females) were assigned to the control group, which were subjected to oral gavages by injecting water every day (1 mL/kg) for 12 weeks. The GBH-treated group was made up of 12 SD rats, 5 males and 7 females, given GBH (100 mg of Gly/kg/day) orally for 12 weeks. The dose was selected from previous reports [41,42] and was adjusted to the rat’s weight weekly. Thereafter, the rats were sedated with pentobarbital sodium, and then 3 mL of blood was drawn from the rats. The rats were then sacrificed by overdose of pentobarbital via intraperitoneal injection. Finally, the rat serum samples were subjected to LC-MS/MS metabolomics analysis. Figure 1 shows the workflow of the study.

### 2.3. Behavioral Evaluation of the GBH Exposed Rats, Barnes Maze

The Barnes Maze test was carried out according to the protocol described by Pitts et al. [43]. Briefly, the paradigm was developed in a testing room, and surrounding visual clues were placed and kept constant during the experiment. The test was developed over 5 days. On day 1, the rats were exposed for 2 min to the maze and the escape tunnel, then the rats were housed in the experimental room for 30 min to decrease the anxiety due to neophobia. The training session started after the rats completed 180 min of habituation. The training sessions were conducted four times a day during four days with a total of 16 assays. The assay started by placing the rat in the middle of the circular illuminated maze covered by a dark bucket, which was hoisted allowing for the rat to explore the maze for up to 2 min or the necessary time to enter the escape tunnel (end of the assay). The rat was exposed to white light and 100 dB noise during the training sessions and the probe trial. Exclusively for assay 1 on day one, the rats that could not find the escape tunnel were gently guided to it and retained for 2 min. The surface of the platform was cleaned with a solution of 70% ethanol to remove odors that could give clues to the next rat tested. A memory test was performed at day five; the escape tunnel was removed and the rats freely explored the maze for one min. Parameters such as errors (wrong holes visited before entering the escape tunnel); latency time (time it took the rats to enter the escape tunnel); and time in the target zone (considered the hole ±1 where the escape tunnel was during the training session) were used to evaluate the rats’ memory. Finally, based on the parameter primary hole distance (number of holes between the first hole visited and the escape tunnel) during the memory test, the spatial recognition index was calculated with the following formula:

(1) $$Spatial\;recognition\;index=\left(\frac{primary\;hole\;distance}{10}\right)\times 100$$

### 2.4. Metabolites Extraction

The polar metabolites were extracted from 100 µL of rat blood serum by modifying previously published method by Want et al. [44], and then transferred into Eppendorf tubes of 1.5 mL. A 200 μL mixture of DCM/MeOH (1:2 *v*/*v*) was added to the samples and vortexed for 30 s. Following a 60 min incubation period at room temperature, 75 μL of DCM was added to the mixture, and the mixture was vortexed for 30 s. An additional 75 μL of cold water was added and vortexed for 30 s. Finally, the samples were centrifuged for 15 min at 5000 rpm. The aqueous phase (upper layer) was then collected and transferred to new Eppendorf tubes. The samples were dried and resuspended in a solution of methanol: water (1:1), then the polar metabolites were subjected to LC-MS/MS analysis. Pooled samples were prepared and injected between the analytical sequence for system verification, and the solution of methanol–water (1:1) was used as a blank.

### 2.5. LC-MS/MS Conditions

The samples were separated and analyzed on an Acquity UPLC HSST3 100 Å (2.1 × 100 mm) column (Waters, Ireland) using a Vanquish UHPLC system (Thermo Scientific, San Jose, CA, USA) coupled to a Quadrupole Exactive HF. Using mobile phase A (MPA) comprising of 0.1% FA in water and mobile phase B (MPB) comprising of 0.1% FA in MeOH, a multistep mobile phase gradient was used. After 20 min at 0.5% MPB, the gradient increased progressively to 50% over the next 5.5 min. The gradient was ramped up to 98% of MPB in 0.5 min and kept steady for 6 min. After that, the column was equilibrated with 0.5% MPB for 2 min. Following the LC separation, a mass spectrometer operated in both positive and negative ion modes was used to analyze the polar metabolites using an analytical ESI source. In the positive ion mode, the spray voltage was set to 3.5 kV, and the transfer tube temperature was set to 300 °C, while 3.0 kV spray voltage was used with the temperature of the transfer tube set at 320 °C in the negative ion mode. The full MS scan was performed using an Orbitrap mass analyzer set at a mass range of 75 to 750 *m*/*z* and resolution of 120,000. An AGC target of 3.0 × 10^6^ and an exclusion list of top 100 intense peaks between 0–6 min was generated from the blank that was added to the full MS method. The scan range number was configured to a value of 1, while the maximum injection duration was set to 200 ms. To generate the MS/MS, an orbitrap scan was acquired in a data-dependent approach, employing a duty cycle of 3 s and the top four precursor ions with the highest intensity were selected for collision-induced dissociation (HCD) MS/MS scan. The scan was conducted using a stepped normalized collision energy (NCE) of 20%, 40%, and 60% for both positive and negative ion modes. Using a Quadrupole isolation mode, with an isolation window of 4 *m*/*z* and a dynamic exclusion of 10 s for the positive ion mode, while a 1 *m*/*z* isolation window and dynamic exclusion of 6 s were set for the negative ion mode. The mass analyzer resolution was set to 30,000 for the positive mode and 15,000 for the negative mode, with a set scan range of 75 to 2000 *m*/*z*, and 50 ms maximum injection time.

### 2.6. LC-PRM-MS Data Validation

A targeted PRM method was employed for the validation of the differentially expressed metabolites (DEMs) between the studied groups. A transition list for the PRM analysis was prepared using the metabolite information acquired from the discovery stage: name, structural formula, *m*/*z* value, and retention time. The gradient that was utilized for the investigation of untargeted metabolomics was not altered in any way. The retention times of the precursors, which were obtained using untargeted proteomics, were manually re-checked using Xcalibur (Thermo Scientific) from the raw data of the pooled sample, and in the end, 178 metabolites were targeted. The PRM data were processed and quantified using Skyline software version 21.2.0.536 for the purpose of quantitative validation, and the normalized data collected were used for the statistical analysis.

### 2.7. Data Analysis

The raw data acquired was analyzed using Compound Discoverer 3.1 software to identify and quantify the metabolomics compositions present in the samples. IBM SPSS 29.0 software was used to perform the Mann–Whitney U test ROC analysis between cohorts. Freestyle 1.4 and Xcalibur 4.2 were used to manually confirm the transition fragment for PRM validation, while Skyline MS 23.1 was used to quantify the PRM data. Origin 2.0 software was used for the principal component analysis (PCA), while the heatmap was generated using Genesis 1.8.1 software, and the boxplot was created with Graph Prism 10.0.2.

## 3. Results

### 3.1. Chronic Exposure to GBH-Induced Alterations in Spatial Memory and Learning

**Figure 2:**
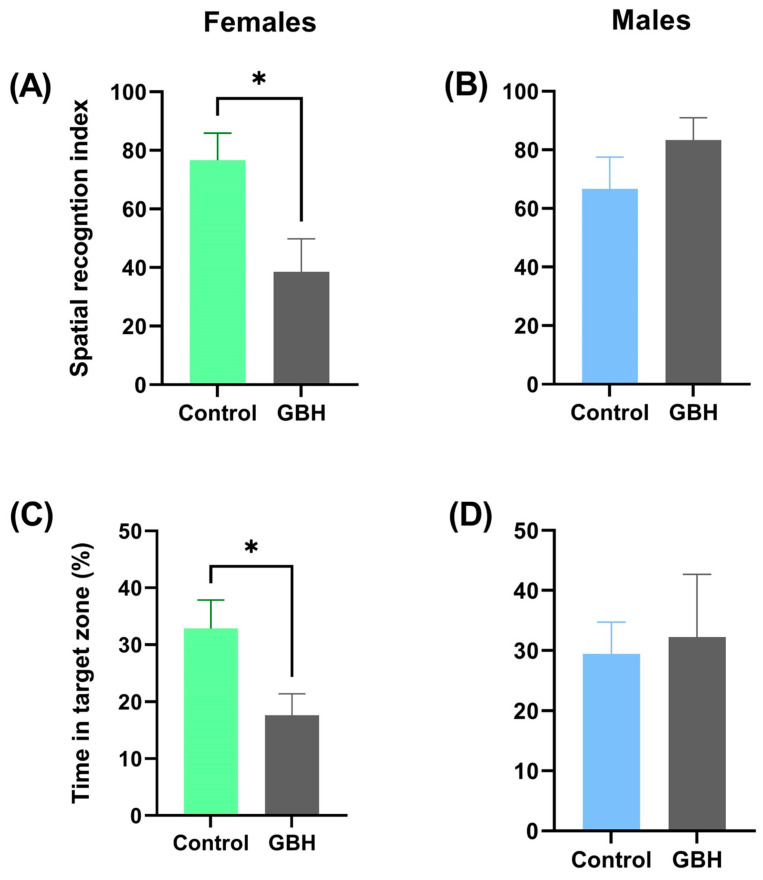
Effect of GBH on the memory on the test day. Spatial recognition index of (**A**) female and (**B**) male rats and time in the target zone of (**C**) female and (**D**) male rats in the Barnes maze (n = 6–7 rats/group). Results are presented as mean ± SEM. * *p* < 0.05.

No changes in spatial navigation strategies to solve the Barnes Maze were detected (*p* > 0.05) in females or males (Supplementary Figure S1A,B) exposed to GBH. Supplementary Figure S2 shows that female rats from the control group showed improved resolution in the Barnes Maze compared to total errors in assay 1 vs. assay 16 (*p* < 0.05), although this improvement was not observed in the latency (*p* > 0.05). In contrast, male rats from the control group dramatically decreased both total errors and latency by comparing assay 1 vs. assay 16 (*p* < 0.05; Supplementary Figure S2). The total errors in female rats and the decrement in total errors and latency in male rats were absent in rats chronically exposed to 100 mg/kg/day GBH (*p* > 0.05). Figure 2 shows the spatial recognition index and time spent in the target zone during the memory test. Female rats chronically exposed to GBH showed a worse spatial recognition index and spent less time in the target zone compared with the control group, see Figure 2A,C; *p* < 0.05. In contrast, no differences were detected (*p* > 0.05) in male rats exposed to GBH compared with the control group, see Figure 2B,D.

### 3.2. Rat Serum Metabolomics Analysis

**Figure 3:**
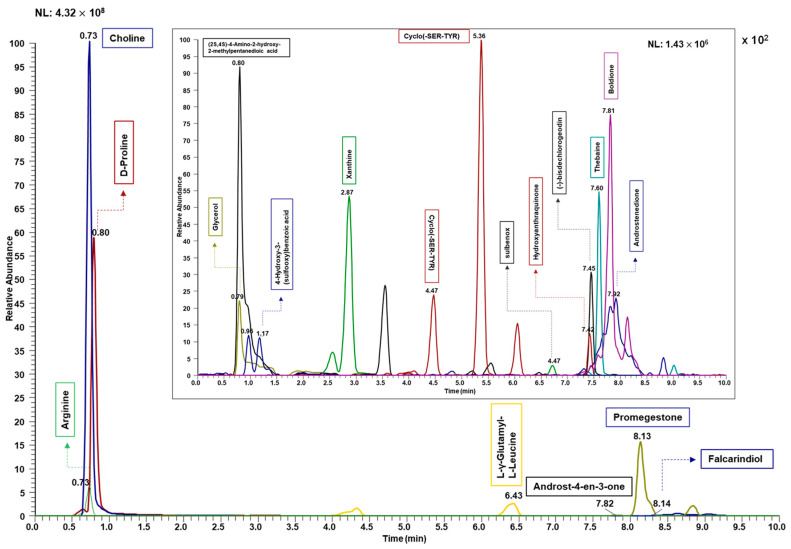
Extracted ion chromatogram (EIC) profile of representative metabolites in the GBH-exposed rat pooled serum samples (n = 24). The data analysis was completed using Compound Discoverer (CD) software from Thermo Sci., and the peaks were manually extracted from the pooled sample raw file using Xcalibur Qual Browser.

Initially, the sera samples derived from the studied rats were subjected to metabolomics analysis. Briefly, the metabolites were extracted with a mixture of dichloromethane–methanol from the sera samples. The samples were dried and reconstituted in a methanol–water solution (1:1) and analyzed in the LC-MS system. A “blank” injection of the dilution solvent was used to create an exclusion list to subtract system background signals from the quantitative results. Additionally, a pooled QC sample was injected across the analytical sequence to validate the precision of the observed results. The data analysis was completed using Compound Discoverer (CD) software from Thermo Scientific. The workflow is described in Figure 1. The Extracted Ion Chromatogram (EIC) of some of the observed metabolites is shown in Figure 3.

### 3.3. Comparative Serum Metabolomics Analysis between Control and GBH-Exposed Rats

**Figure 4:**
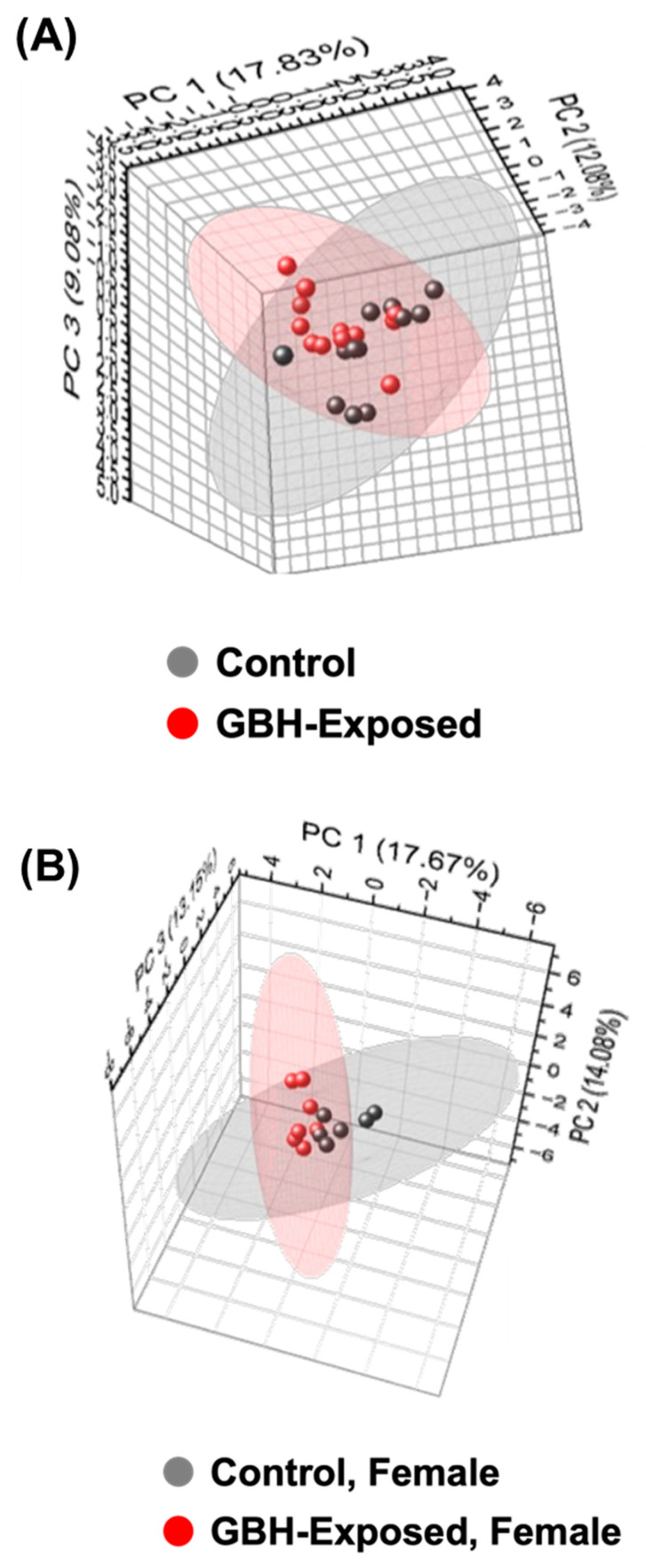

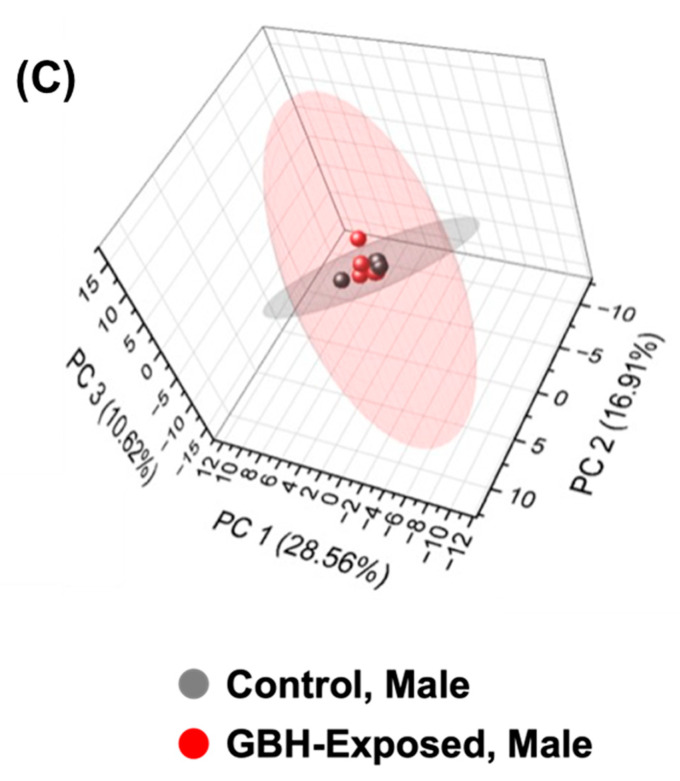
Principal component analysis (PCA) generated with a confidence level of 95%; (**A**) control and GBH-exposed groups comparing the whole data set (n = 24); (**B**) comparison of the female samples from the studied cohorts (n = 13); and (**C**) comparison of the male samples from the studied cohorts (n = 11). The result shows that the female group plot produced a better group clustering of the different groups separation than the other comparisons.

Using Compound Discoverer, we identified 1165 compounds across the tested serum samples. We then manually searched through the Human Metabolome Database (HMBD), KEGG Compound Database, and PubChem Database for metabolite IDs for the identified compounds. After the search, we mapped 965 metabolites to their IDs, and these metabolites were further analyzed. Principal component analysis (PCA) was used to visualize the data differences between the investigated sample groups. Figure 4 shows the PCA plot derived from the identified metabolites. The PCA was generated with a confidence level of 95%. The PCA plots generated with the whole control/GBH-exposed data sets showed low group separation, see Figure 4A. The PCAs comparing the female and male groups were plotted in Figure 4B,C. The result shows that the female group plot produced a better group separation than the other comparisons.

### 3.4. Differentially Expressed Metabolites (DEMs)

The ability of individual metabolites to differentiate the control and GBH-exposed serum rat samples was investigated using the Mann–Whitney U test with 95% confidence (*p* value < 0.05). Of the 965 analyzed metabolites, 117 were statistically significant in their expression. But after the Benjamini–Hochberg correction, 70 metabolites were statistically significant (*p* value < 0.05) (Supplementary Table S2). When the samples were separated and compared based on their gender (male or female), 107 and 50 metabolites showed statistical significance in their expression in the female and male groups, respectively. After the Benjamini–Hochberg correction, 98 and 1 metabolites were statistically significant (*p* value < 0.05) in the female and male cohorts, respectively (Supplementary Table S3).

A Venn diagram investigating the common and unique DEMs in the studied cohorts and the gender groups is shown in Supplementary Figure S3A. A total of 192 DEMs were observed across all comparisons, with only 2 DEMs overlapping in all the comparisons. There were 2 metabolites commonly observed to be significant in both male and female groups, while 51 metabolites were unique to the female and 22 metabolites to the male subgroup.

Heatmaps were used to visualize and compare the serum abundances of the DEMs across the control and GBH-exposed rat groups. Supplementary Figure S4 shows the metabolite-specific heatmap for the combined and the gender groups, revealing that the metabolome of the GBH-exposed serum samples has differentially expressed metabolites that can be further investigated. In the combined group, 41 DEMs were observed to be downregulated in GBH-exposed rats, while 76 metabolites were upregulated. In the gender groups, 21 and 32 DEMs were observed to be downregulated in the GBH-exposed male and female groups, respectively, while 32 and 75 metabolites were upregulated in the male and female groups, respectively. The downregulated metabolites had relatively low levels of expression in GBH-exposed rats, while the upregulated metabolites were observed to have a higher level of expression in GBH-exposed rats. This is consistent with the calculated fold change in all the DEMs. The range of visualization is from green (−3.0) to red (3.0), depicting the level of metabolome changes between GBH-exposed and healthy control samples.

### 3.5. PRM Validation of DEMs

After the DEMs identification, we validated their expression between the analyzed cohorts using PRM target analysis. The validated DEMs maintained the same trend in fold change when the identified and quantified metabolomics study was carried out. We validated 36 metabolites in the combined group and 47 metabolites in the female groups to follow the same trend in the PRM as observed in the initial full scan. The PRM transition list, including the transition *m*/*z*, fragment ions, fold changes, and log2 of fold change for the evaluated metabolites, is described in Supplementary Tables S4 and S5.

### 3.6. Ingenuity Pathway Analysis (IPA)

**Figure 5:**
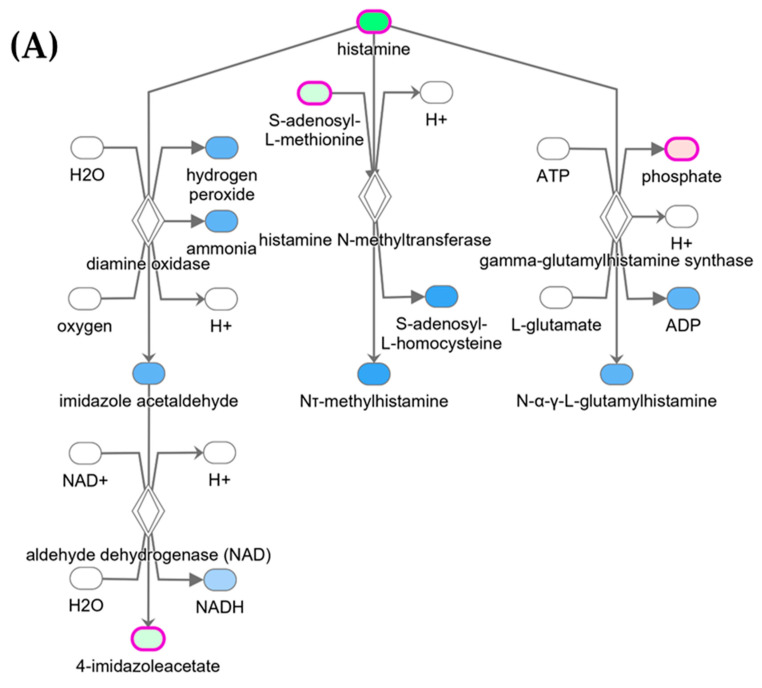

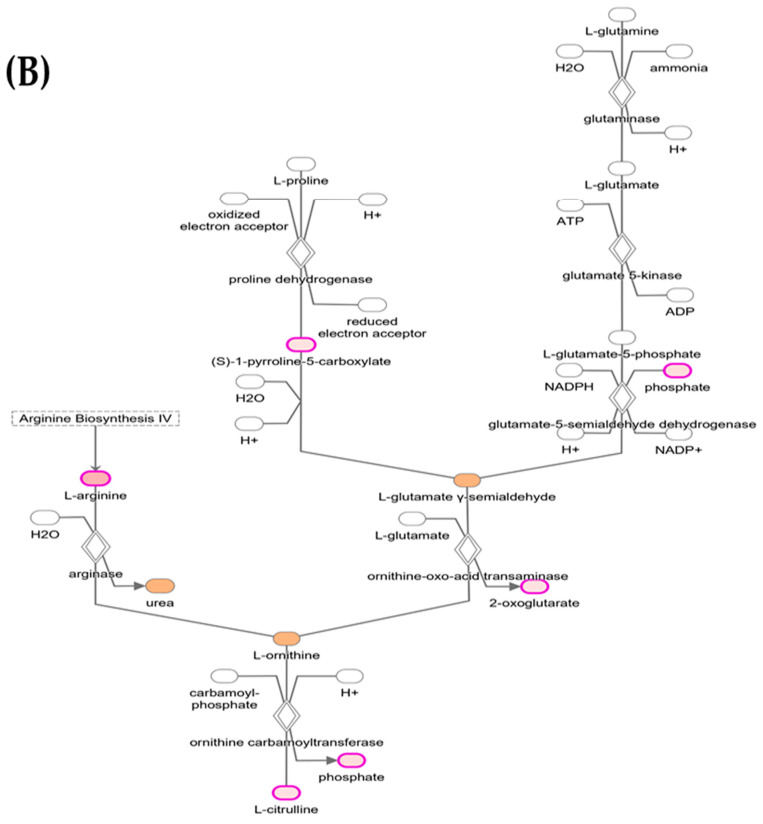
Ingenuity pathway analysis (IPA). (**A**) Activation of the histamine degradation pathway showing *z*-score of 1.00 and *p*-value of 0.02, and (**B**) activation of the citrulline biosynthesis pathway showing *z*-score of 1.00 and *p*-value of 0.02. (The red color indicates increased measurement; green represents decreased measurement; blue represents predicted inhibition; and orange represents predicted activation).

To gain more understanding about the impacts of the changes in the metabolome expressions on cellular functions, pathway analysis was performed using IPA (QIAGEN Bioinformatics). The logarithm of fold changes and *p*-values of all metabolites were uploaded to IPA software version 107193442. Using *p*-values and *z*-scores, IPA mapped and clustered the metabolites to the canonical pathways, as well as identified the correlations and connections of biological activities and diseases to other metabolites. The importance of each clustering behavior is described using the *p*-value of the IPA, and the *z*-score is used to infer the activation statuses of putative regulators (upstream) and functions/pathways (downstream). The result of the IPA showed 35 different pathways, including the inhibition of the histamine degradation pathway and the activation of the citrulline biosynthesis pathway, both showing a *z*-score of 1.00 and *p*-value of 0.02 (Figure 5A,B).

The results of the IPA prediction show a relationship between 8 DEMs (Table 1) and some important diseases and functions derived from the activation or pathway predictions. The most important were neurodegenerative diseases, cognitive disfunction, urination disorder, disruption of the blood–brain barrier, depression, and mood disorder. Some of these important disease pathways are shown in Supplementary Figure S5.

### 3.7. Dot Plots and ROC/AUC Values of the Most Important DEMs

**Figure 6:**
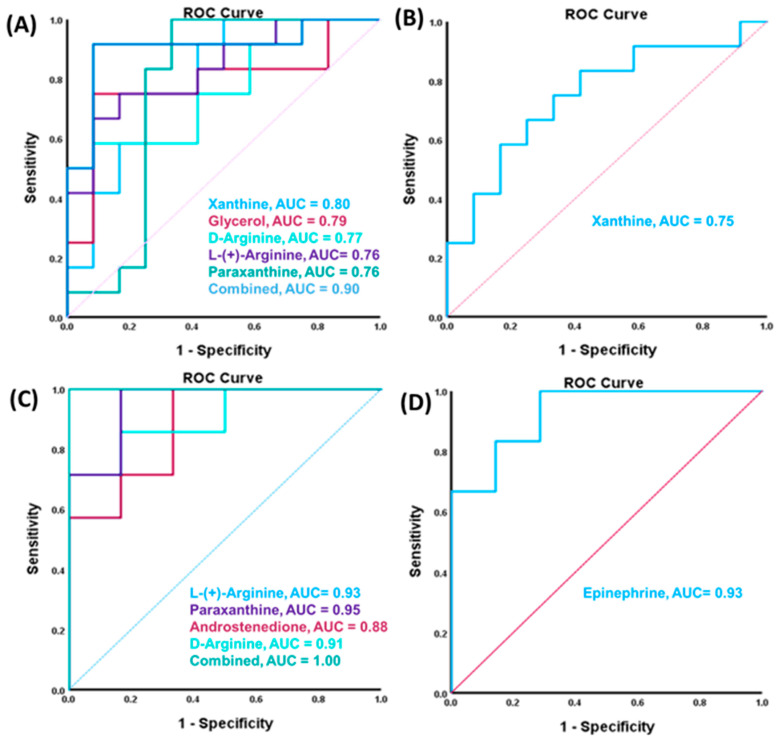
ROC/AUC curves of the DEMs shown to be implicated in some important diseases and functions in the (**A**) combined group ROC curve for downregulated metabolites, (**B**) combined group ROC curve for upregulated metabolites, (**C**) female group ROC curve for downregulated metabolites, and (**D**) female group ROC curve for upregulated metabolites. The significant metabolites in the female rat subgroup showed high AUC value scores and a combined AUC score of 1.00.

To investigate the ability of the most important DEMs to differentiate between the studied groups, dot plots and receiving operating characteristic (ROC) curves were created. The boxplots in Supplementary Figure S6 show the changes in DEMs predicted to be implicated in the progression of the described diseases in Table 1 from the healthy control to GBH-exposed groups. Notably, alterations in the metabolites were more pronounced in the female group with most of the significant metabolites implicated in several diseases, which correlated with our observations of the spatial learning of the GBH-exposed rats. The ROC curves were performed for the eight most important DEMs (Figure 6); their *p*-values, fold change, and AUC values are shown in Supplementary Table S6. For the combined group, downregulated metabolites xanthine, paraxantine, glycerol, D-arginine, and L-(+)-arginine with AUC values of 0.80, 0.76, 0.79, 0.77, and 0.76, respectively (Figure 6A). The single metabolite with upregulation identified in the combined group was xanthine with an AUC value of 0.75, see Figure 6B. The evaluation of the ability to differentiate the studied cohorts of the metabolites was also revised by gender. The downregulated metabolites in the female group were paraxanthine, androstenedione, D-arginine, and L-(+)-arginine with AUC values of 0.95, 0.88, 0.91, and 0.93, respectively, while the single upregulated metabolite in the female group was epinephrine with an AUC value of 0.93. Also, the combined AUC value was calculated for these significant metabolites in both the combined rat group and female subgroup to give AUC values of 0.90 and 1.00, respectively. The result of the ROC analysis for most metabolites gave considerably large AUC values in the female rat subgroup compared to the other evaluations. Some of the observed metabolites with significantly high scores include paraxanthine (0.95), epinephrine (0.93), L-(+)-arginine (0.93), and D-arginine (0.91).

## 4. Discussion

Due to the widespread use of glyphosate and GHB-based herbicides, glyphosate residues in crops have increased to levels that call for serious attention. For many years, glyphosate has been regarded as harmless or minimally toxic to mammals due to the absence of its primary target, the shikimic acid pathway in humans [2]. Nonetheless, mounting evidence suggests that glyphosate may cause adverse health effects in humans via other mechanisms, thus requiring thorough investigation [5]. Likewise, multiple epidemiological studies have also shown that chronic exposure to GBH can be harmful to human’s health [45,46,47]. Glyphosate and exposure to GBH have been linked to neurobehavioral changes that stem from the impairment of neuronal developmental processes in mice [8]. In this study, we completed the serum metabolome profile of rats after their exposure to chronic GBH. The main aim was the identification of a possible link of the metabolome changes to neurophysiologic, deleterious, and other diseases in the exposed rats. Thus, we investigated the metabolome changes in both male and female rats for a better understanding of how these changes vary between genders. From the 965 metabolites identified in 24 serum rat samples, we identified 71 DEMs after Benjamini–Hochberg correction, with 7 downregulated and 64 upregulated. We further separated the samples into male and female gender groups where 1 and 96 metabolites were seen to show statistical significance in their expression after Benjamini–Hochberg correction, respectively. The PRM assay validated the expression of 37 DEMs in the combined group, while 46 metabolites were validated in the female rat group. The impacts of the changes in metabolomic expressions on cellular functions were observed by performing the IPA analysis. The result of the IPA showed 35 different implicated pathways, including the histamine degradation and citrulline biosynthesis pathways, which showed significant activation with a *z*-score of 1.0 and *p*-value of 0.02. Histamine, a neurotransmitter produced by specialized brain cells called histaminergic neurons, plays a crucial role in regulating various physiological functions such as sleep, appetite, learning, arousal, energy, metabolism, and immune response [48]. Histamine is typically associated with peripheral allergic and inflammatory responses, but it can also regulate brain inflammation [49] and neurogenesis [50]. It is capable of promoting both inflammatory and regulatory responses, both of which contribute to pathological processes [49]. Reduced histamine levels can result in neurobehavioral symptoms such as learning and memory difficulties, as well as anxiety-related behaviors [51,52]. Numerous neurological and psychiatric disorders have been linked to changes in histaminergic neurotransmission. This includes mood and sleep disorders, eating disorders, epilepsy, cognitive disorders, addiction, movement disorders, pain, and neuroinflammation [53]. The observed activation of the citrulline biosynthesis pathway from the IPA result could come from the functional response of the body to mitigate the effect of the neurological damage and other changes in the rat metabolites due to the chronic exposure of GBH. Some antioxidants such as glutathione has been shown to maintain physiological homeostasis and metabolism, playing a vital role in neuronal defense through the use and regulation of reactive oxygen and oxygen and nitrogen species [54]. Citrulline has high antioxidant effects, which may, at least in part, explain the preventive impact that supplementation with citrulline has on age-related LTP decline [55]. In the nitric oxide cycle, the neutral amino acid L-citrulline serves as a precursor to L-arginine. L-citrulline has recently been revealed to have a neuroprotective effect to alleviate cerebrovascular dysfunction by preventing neuronal cell death and protecting against cerebrovascular damage, and it is known to prevent age-related long-term potentiation (LTP) decline in old rats [56].

Research indicates that the entry of toxic substances into the brain triggers glial cells [57], and a reactive state of glia referred to as gliosis is a pathological hallmark of all types of central nervous system injuries [58,59]. Microglia and astrocytes, which are present in all cases of neuroinflammatory response, have been shown to be involved in the development of neurotoxicity caused by toxic substances and the advancement of neurodegeneration in many neurological disorders [59,60,61]. The neurotoxic effects of GBHs in preclinical reports include slight alterations in cognition (e.g., attention, learning and memory, etc.), especially after chronic exposure in adult subjects [62,63]. Our behavioral study suggests that both female and male rats exposed to GBHs showed an interference with learning by prevention of the decrease in latency and error. These results are in agreement with those obtained on the Morris water maze employing glyphosate on pups of Wistar rats exposed to 35 or 70 mg/kg/day for 20 days [64], showing impairments in learning and spatial memory. Regrettably, their study did not indicate if the behavioral study was performed in male or female rats, but our findings suggest a higher susceptibility in female population to spatial memory alterations. Interestingly, the alterations in spatial learning and memory induced by GBHs both in female and male animals did not alter the strategies used to solve the Barnes maze. Thus, it is possible that the alterations induced by GBHs reach specific brain nucleus of neuron populations related with spatial memory, but not with spatial navigation. Clearly, further, and deeper experiments are needed to explore this speculation. The significant activation of the histamine degradation pathway in the rat samples because of chronic exposure to GBH suggests that exposure to GBH could lead to numerous neurological and psychiatric conditions, which explains the alterations in spatial memory and learning observed in the exposed rats. Studies by Yamada et al. [65] have demonstrated that chronic depletion of brain histamine in adult mice induces depression-like behaviors and impairs the sleep–wake cycle because of the involvement of histamine in promoting wakefulness and maintaining the circadian rhythm [1].

Furthermore, we identified eight observed DEMs that were significantly involved in some important diseases and functions, including neuromuscular diseases, inflammation of organs, cognition, and urination disorder after glyphosate exposure. Hence, the ROC curve analysis was performed for these DEMs in both the combined and gender groups to determine their sensitivity and specificity in differentiating between the control and the GBH-exposed group. The result of this analysis indicated AUC values of 0.75 for downregulated serum metabolites in the combined rat group, and the upregulated metabolites showed a combined AUC value of 0.93. When we considered the gender of the rats, the downregulated metabolites in the female rats’ serum showed large AUC values of 0.93 and the upregulated metabolites showed a combined AUC score of 1.00. These changes in the important DEMs in both comparisons were visualized using boxplots to show how these metabolites increased or decreased between the control and the GBH-exposed group. Some of the important metabolites observed to be involved in the neurophysiological changes in the GBH-exposed rats with excellent AUC values include the downregulated epinephrine (AUC = 0.93), as well as upregulated L-(+)-Arginine (AUC = 0.91) and D-Arginine (AUC = 0.93). Notably, the female group showed the most significant changes compared to the male subgroup and the combined group, which correlates with the behavioral evaluation we observed in the spatial recognition index and the time spent in the target zone during the memory test. The female rats chronically exposed to GBH showed a worse spatial recognition index and spent less time in the target zone compared with the control group and, in contrast, no differences were detected in male rats exposed to GBH compared with the control group. This is indicative of major damage in the female groups compared to the control after exposure to GBH, which could also be responsible for the changes in their serum metabolites.

Interestingly, we observed a downregulation in epinephrine, and the IPA result reveals the implication of this DEM in neuromuscular diseases, inflammation of organs, cognition, and urination disorder among others. Epinephrine (adrenaline) is a neurotransmitter (chemicals that transmit signals between nerve cells) and a hormone which plays an important role in the body’s fight-or-flight response. Low levels of epinephrine have been reported to result in physical and mental symptoms, such as anxiety, depression, and changes in blood pressure [66]. The impaired synthesis of epinephrine is associated with autosomal recessive neurodevelopmental disorder [67]. There is, therefore, an indication that the downregulation in epinephrine is probably linked to the development of neurological disorders after exposure to GBH in the female rat samples. The observed upregulated paraxanthine in GBH-exposed rats is seen to be involved in the inhibition of neurodegeneration of dopaminergic neurons and our IPA result showed no prediction for this disease condition. There is a possibility that the increased level of paraxanthine after glyphosate exposure is a response to inhibit the neurodegeneration of the dopaminergic neuron. This suggests that exposure to GBH could result in the deterioration of the dopaminergic neuron, a condition associated with development of Parkinson’s Disease (PD) [68]. Similarly, we observed an upregulation in L-(+)-arginine and D-arginine, metabolites predicted to be associated with the formation of reactive oxygen species (ROS). Increased levels of reactive oxygen species (ROS) inside cells can be harmful because they affect the biomolecular structure of cellular macromolecules including proteins, lipids, and nucleic acids, which can in turn impair the normal operations of tissues and organs [69]. ROS can oxidize the sulfur in a protein’s structure, which can prevent the protein from folding properly and contribute to the development of age-related conditions like PD and Alzheimer’s disease (AD) [70,71,72]. Exposure to toxins in the environment including pesticides, herbicides, and solvents has been linked to PD-associated risk [73,74]. Specifically, studies have also shown that rural living and agricultural jobs have been touted as significant risk factors for developing PD, which appears to be chiefly due to an increased exposure to pesticides [75,76]. Similarly, research has indicated that the recent rise in use of glyphosate on soy and corn crops correlates with a notable upsurge in mortality rates attributed to Alzheimer’s disease and other neurological disorders [74].

The IPA report predicted that the reduced level of epinephrine and increased level of L-arginine would contribute to the activation of urination disorder in the GBH-exposed rats. Chang et al. [77], in their study, showed that there is a possible relationship between glyphosate exposure and urinary oxidative stress in farmers. Urination disorder has been reported to be associated with other health problems such as aging, bladder infection, blocked urinary tract from a tumor or kidney stone, and diabetes [78]. Bladder nerves and muscles have also been reported to be damaged or affected by the development of neurodegenerative diseases [79,80,81]. Loss of neurogenic bladder control is caused by the selective degeneration of dopaminergic neurons in the substantia nigra and potentially also in the ventral tegmental region in people with PD. This degeneration leads to disruption in a complex network, which results in the loss of selective disinhibition of bladder reflexes [81,82]. In typical parkinsonism, including multiple system atrophy, lower urinary tract symptoms are extremely common, and their onset relative to other autonomic and motor symptoms may serve as a diagnostic marker [82]. Therefore, the urination disorder in the GBH-exposed rats could be another indication of possible neurophysiological changes due to their chronic exposure to GBH.

## 5. Conclusions

In conclusion, the findings from this present study demonstrate that GBH exposure can alter the spatial memory and the expression of some important metabolites that are linked to neurophysiological disorders in young rats, with the female rats showing higher susceptibility compared to the males. This indicates that the female rats are more affected by the exposure than the males, and that they are more likely to show early symptoms of neurophysiological disorders with chronic exposure to GBH. Four important metabolites (paraxanthine, epinephrine, L-(+)-arginine, and D-arginine) were observed to show significant changes and involvement in neurological alterations as suggested by our IPA result. Hence, in this study our results indicate that chronic exposure to GBH can increase the risk for developing neurological disorders.

## Figures and Tables

**Table 1:** List of DEMs shown to be implicated in some important diseases and functions based on IPA result.

DEMs	Implicated Diseases
Paraxanthine ^1,2^	Neurodegeneration of dopaminergic neurons, Necrosis
Epinephrine ^2^ 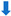	Inflammation of organ, Cognition, Neuromuscular disease, Urination disorder, Necrosis
D—Arginine ^1,2^ 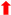	Formation of reactive oxygen species
L—(+)-Arginine ^1,2^ 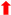	Formation of reactive oxygen species
Choline ^1^ 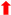	Inflammation of organ, Progressive neurological disorder, Apoptosis, Necrosis
Xanthine ^1^ 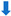	Neurodegeneration of dopaminergic neurons, Necrosis
Glycerol ^1^ 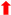	Inflammation of absolute anatomical region, Disruption of blood–brain barrier, Urination disorder, Necrosis
Androstenedione ^2^ 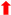	Depressive disorder, Mood disorders

Statistically significantly metabolites in the combined group ^1^, the female group ^2^ (Upregulated: 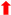, Downregulated: 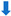).

## Data Availability

The raw data are available on MetaboLights [83] with accession number MTBLS8995 (www.ebi.ac.uk/metabolights/MTBLS8995).

## Supplementary Materials

The following supporting information can be downloaded at: https://www.mdpi.com/article/10.3390/metabo14010050/s1, Supplementary Table S1: Details on the solvents and reagents used. All solvents used for this experiment were HPLC-grade.; Supplementary Table S2: Metabolites with significant changes in abundance between control and GBH-exposed groups in the combined groups; Supplementary Table S3: Metabolites with significant changes in abundance between control and GBH-exposed groups in the gender subgroups; Supplementary Table S4: List of DEMs in the combined rat group validated by LC-PRM-MS including *m*/*z*, and FC before and after PRM validation; Supplementary Table S5: List of DEMs in the female rat subgroup validated by LC-PRM-MS including *m*/*z*, and FC before and after PRM validation; Supplementary Table S6: List of DEMs shown to be implicated in some important diseases and functions based on IPA results; Supplementary Figure S1. Effect of chronic exposure to GBH on search strategy of (A) females and (B) males studied on Barnes maze (n = 6–7 rats/group); Results are presented as mean ± SEM; Supplementary Figure S2. x Effect of chronic exposure to GBH on (A and B) total errors and (C and D) latency to solve the Barnes maze in rats (n = 6–7 rats/group). Results are presented as mean ± SEM. * *p* < 0.05; Supplementary Figure S3. Venn shows overlapping and unique significant metabolites in the whole data set and gender comparison between control and GBH-exposed rats. The result shows two common metabolites in all comparisons, and female rats showed the most unique metabolites. Supplementary Figure S4; Heatmap of significant metabolites in (A) control and GBH-exposed groups in the combined sample group, (B) female rat samples, and (C) male rat samples (C_M = Control (male); C_F = Control (female); 100_M = GBH-exposed (male); 100_F = GBH-exposed (female)). The red color indicates upregulated while the green color indicates downregulated metabolites; Supplementary Figure S5. (A) Canonical pathways, (B) Pathways network, (C) Prediction legends. (Red indicates increased measurement, Green represents decreased measurement, Blue represents predicted inhibition, and Orange represents predicted activation); Supplementary Figure S6: Boxplot showing the changes in the DEMs from combined group comparison between control to GBH-exposed. Five metabolites were upregulated, and one metabolite was downregulated; Supplementary Figure S7. Boxplot showing the changes in the control and GBH-exposed DEMs in female rats. Four metabolites were upregulated, and one metabolite was downregulated.
