# Supplementary material for: Metabolomic Changes in Rat Serum after Chronic Exposure to Glyphosate-Based Herbicide
Source: Metabolites. 2024 Jan 13;14(1):50. doi: 10.3390/metabo14010050 (PMC10819816; doi:10.3390/metabo14010050)
Supplement: Supplementary file 1 [file metabolites-14-00050-s001.zip › metabolites-2808183-supplementary.pdf]

[Supplementary data]

Metabolomics Changes in Rat Serum after Chronic Exposure to Glyphosate-Based Herbicide

Oluwatosin Daramola[1‡], Cristian D. Gutierrez Reyes[1‡], Jesús Chávez-Reyes[2], Bruno A. Marichal-Cancino[2], Judith Nwaiwu[1], Sherifdeen Onigbinde[1], Moyinoluwa Adeniyi[1], Joy Solomon[1], Md Mostofa Al Amin Bhuiyan[1], and Yehia Mechref[1*]

1. Department of Chemistry and Biochemistry, Texas Tech University, Lubbock, TX 79409, USA.
2. Departamento de Fisiología y Farmacología, Centro de Ciencias Básicas, Universidad Autónoma de Aguascalientes, México.

*Corresponding author
Department of Chemistry and Biochemistry
Texas Tech University
Lubbock, TX 79409-1061
Email: yehia.mechref@ttu.edu
Tel: 806-742-3059
Fax: 806-742-1289

‡ These authors contribute equally

Table of Contents:

Supplementary Tables:

Supplementary Table S1: Details on the solvents and reagents used. All solvents used for this experiment were HPLC-grade.

Supplementary Table S2. Metabolites with significant changes in abundance between control and GBH-exposed groups in the combined groups.

Supplementary Table S3: Metabolites with significant changes in abundance between control and GBH-exposed groups in the gender subgroups.

Supplementary Table S4: List of DEMs in the combined rat group validated by LC-PRM-MS including m/z and FC before and after PRM validation.

Supplementary Table S5: List of DEMs in the female rat subgroup validated by LC-PRM-MS including m/z and FC before and after PRM validation.

Supplementary Table S6: List of DEMs shown to be implicated in some important diseases and functions based on IPA results**.**

Supplementary Figures:

Supplementary Figure S1. Effect of chronic exposure to GBH on search strategy of (A) females and (B) males studied on Barnes maze (n = 6 - 7 rats/group). Results are presented as mean ± SEM.

Supplementary Figure S2. Effect of chronic exposure to GBH on (A and B) total errors and (C and D) latency to solve the Barnes maze in rats (n = 6 - 7 rats/group). Results are presented as mean ± SEM. *$p < 0.05$.

Supplementary Figure S3. Venn shows overlapping and unique significant metabolites in the whole data set and gender comparison between control and GBH-exposed rats. The result shows two common metabolites in all comparisons, and female rats showed the most unique metabolites.

Supplementary Figure S4. Heatmap of significant metabolites in **A)** control and GBH-exposed groups in the combined sample group, **B)** female rat samples, and **C)** male rat samples (C_M = Control (male); C_F = Control (female); 100_M = GBH-exposed (male); 100_F = GBH-exposed (female)). The red color indicates up regulated while the green color indicates down regulated metabolites.

Supplementary Figure S5. A) Canonical pathways, B) Pathways network, C) Prediction legends. (Red indicates increased measurement, Green represents decreased measurement, Blue represents predicted inhibition, and Orange represents predicted activation).

Supplementary Figure S6: Boxplot showing the changes in the DEMs from combined group comparison between control to GBH-exposed. Five metabolites were up regulated, and one metabolite was down regulated.

Supplementary Figure S7. Boxplot showing the changes in the control and GBH-exposed DEMs in female rats. Four metabolites were up regulated, and one metabolite was down regulated.

Supplementary Table S1: List of all identified metabolites**.**

Supplementary Table S1: Details on the solvents and reagents used. All solvents used for this experiment were HPLC-grade.

Solvents/Reagents	Catalog number	Lot number	Concentration Used	Vendor
Methanol	A412-4	232680	100%	Fisher Scientific (Fair Lawn, NJ)
Acetonitrile	A998SK-4	220848	100%	Fisher Scientific (Fair Lawn, NJ)
Dichloromethane	D143-4	223401	100%	Fisher Scientific (Fair Lawn, NJ)
Water	W5-4	231099	100%	Fisher Scientific (Fair Lawn, NJ)
Formic Acid	A117-50	225582	100%	Fisher Scientific (Fair Lawn, NJ)
Glyphosate	-	DZRL0507	100 mg/day/rat	Rival® herbicide from Monsanto (St. Louis, MO, USA).
Pentobarbital sodium	-	23105SP	0.063 g/mL	Pets Pharma Ltd. (Mex. Mexico)

Supplementary Table S2: Metabolites with significant changes in abundance between control and GBH-exposed groups in the combined groups.

Metabolites	Average (Control) (%)	Average (GBH-exposed) (%)	p-value	Adjusted p-value	Fold Change
(+/-)-6-Hydroxy-3-oxo-alpha-ionone	0.002±0.002	0.004±0.003	0.02	0.04	2.00
Dibenzothiophene	0.008±0.005	0.02±0.008	0.002	0.04	2.30
(Z,E)-9,12-Tetradecadienyl acetate	0.0008±0.0006	0.003±0.003	0.003	0.04	3.45
Thiomorpholine3-carboxylate	0.006±0.002	0.01±0.004	0.003	0.04	1.78
(9S_10S)-10-Hydroxy-9-(phosphonooxy)octadecanoate	0.006±0.003	0.003±0.002	0.003	0.04	0.55
Diethyl phthalate	0.007±0.002	0.01±0.005	0.003	0.04	1.70
Rishitin	0.001±0.001	0.002±0.0006	0.003	0.03	1.48
Laurilsulfate	0.03±0.03	0.07±0.02	0.004	0.04	2.15
Methyl 3,5-ditert-butyl-4-hydroxybenzoate	0.003±0.0009	0.002±0.0007	0.005	0.04	0.68
Abieticacid	0.0003±0.0003	0.006±0.01	0.006	0.04	21.78
L-(+)-Arginine	0.05±0.05	0.1±0.1	0.006	0.04	3.03
(9S_10S)-9_10-Dihydroxyoctadecanoate	0.001±0.0005	0.002±0.0007	0.007	0.04	1.75
2,3,4,5-tetrachloro-4'-biphenylol	0.002±0.0004	0.003±0.0005	0.007	0.04	1.24
Androst-4-en-3-one	0.0006±0.0003	0.004±0.006	0.007	0.04	6.57
Falcarindiol	0.002±0.0009	0.003±0.001	0.007	0.04	1.44
(2E)-2-(hydroxymethyl)-3-[3-oxo-5-(propan-2-yl)-1,3,4,5,6,7-hexahydro-2-benzofuran-4-yl]prop-2-enoic acid	0.005±0.002	0.003±0.002	0.008	0.04	0.54
Glycidyl Stearate	0.004±0.002	0.002±0.001	0.008	0.04	0.45
(2S)-Flavan-4-ol	0.0002±0.0002	0.004±0.008	0.009	0.04	18.45
Deoxycholic Acid	0.03±0.01	0.01±0.007	0.009	0.04	0.50
Rehmaionoside C	0.003±0.002	0.001±0.0007	0.009	0.04	0.50
Sulbenox	0.0001±0.00007	0.0005±0.0009	0.01	0.04	3.98
[FA(20:4)]17R_18S-epoxy-5Z_8Z_11Z_14Z-eicosatetraenoicacid	0.01±0.02	0.02±0.01	0.01	0.04	1.52
Choline	1±0.2	1.2±0.2	0.01	0.04	1.20
[FA(20:4)]5Z_8Z_11Z_14Z-eicosatetraenoicacid	0.009±0.01	0.03±0.02	0.02	0.04	2.82
1,2-dihydroxyheptadec-16-yn-4-yl acetate	0.002±0.001	0.01±0.008	0.02	0.04	4.14
Forskolin	0.0004±0.0004	0.0009±0.0006	0.02	0.04	2.36
L-N2-(2-Carboxyethyl)arginine	0.003±0.003	0.007±0.004	0.02	0.04	2.24
L-Octanoylcarnitine	0.0002±0.0004	0.0006±0.0006	0.02	0.04	2.53
VIRILON	0.0007±0.0003	0.001±0.0004	0.02	0.04	1.52

Metabolites	Average (Control) (%)	Average (GBH-exposed) (%)	p-value	Adjusted p-value	Fold Change
3alpha_11beta-Dihydroxy-5alpha-androstane-17-one	0.0009±0.002	0.002±0.001	0.02	0.04	2.12
5-Ethyl-3,8-dimethyl-1,7-dihydroazulene	0.002±0.001	0.002±0.0007	0.02	0.04	1.30
9-cis-Retinoicacid	0.004±0.002	0.02±0.03	0.02	0.04	5.07
Dihydrokavain	0.0003±0.0007	0.0003±0.0003	0.02	0.04	1.05
Glycerol	0.003±0.001	0.005±0.002	0.02	0.04	1.61
4-Hydroxy-3-(sulfooxy)benzoic acid	0.003±0.004	0.008±0.007	0.02	0.04	2.87
dehydroretinaldehyde	0.002±0.0009	0.004±0.002	0.02	0.04	1.71
Desonide	0.002±0.004	0.003±0.002	0.02	0.04	1.27
N_N-Dimethylsphing-4-enine	0.0002±0.00007	0.0007±0.001	0.02	0.04	3.62
N-Carbamoylputrescine	0.0004±0.0003	0.0008±0.0005	0.02	0.04	2.09
(2E)-2-Tridecene-4,6,8-triyn-1-ol	0.007±0.003	0.01±0.004	0.02	0.04	1.43
D-Arginine	0.006±0.005	0.01±0.008	0.02	0.04	2.10
Promegestone	0.3±0.2	0.4±0.1	0.02	0.04	1.47
Ubiquinone-1(CoQ1)	0.002±0.001	0.04±0.1	0.02	0.04	22.09
1_7-Dimethylxanthine(paraxanthine)	0.0005±0.0007	0.0006±0.0006	0.03	0.04	1.23
3-hydroxy-3-methylpentanedioic acid	0.02±0.01	0.03±0.03	0.03	0.04	2.05
9_12-Dioxododecanoicacid	0.001±0.0004	0.002±0.0004	0.03	0.04	1.29
cis-3-Hexenyl phenylacetate	0.008±0.003	0.01±0.01	0.03	0.04	1.96
ibufenac	0.0002±0.0001	0.0009±0.001	0.03	0.04	4.10
oxeladin	0.0007±0.0004	0.001±0.0006	0.03	0.04	1.63
12-oxo Phytodienoic Acid	0.004±0.002	0.006±0.003	0.03	0.04	1.62
2-Dodecylbenzenesulfonic acid	0.1±0.07	0.2±0.09	0.03	0.04	1.91
5-Methylcytosine	0.06±0.02	0.08±0.03	0.03	0.04	1.39
5-O-methyl embelin	0.004±0.002	0.004±0.001	0.03	0.04	1.15
Benzylideneacetone	0.007±0.003	0.01±0.004	0.03	0.04	1.43
chavicol	0.02±0.002	0.02±0.004	0.03	0.04	1.16
DAPSONE HYDROXYLAMINE	0.001±0.003	0.006±0.008	0.03	0.04	4.81
Michler's ketone	0.00003±0.00004	0.001±0.004	0.03	0.04	43.07
R.g.-Keto III	0.009±0.006	0.01±0.008	0.03	0.04	1.54
19-Oxoandrost-4-ene-3_17-dione	0.0003±0.0002	0.0009±0.0009	0.04	0.04	3.35
2,6-Di-iso-propylnaphthalene	0.002±0.0009	0.003±0.001	0.04	0.04	1.56
235BBF3K97	0.001±0.0004	0.002±0.0007	0.04	0.04	1.43
3-oxopalmitic acid	0.001±0.0004	0.002±0.0004	0.04	0.04	1.28
Auraptene	0.0004±0.0002	0.0008±0.0007	0.04	0.04	2.07
D-Glutamate	0.07±0.03	0.1±0.06	0.04	0.04	1.58
Diethanolamine	0.009±0.009	0.05±0.07	0.04	0.04	5.33
1,3-Dihydroxy-2-propanyl (9Z)-9-tetradecenoate	0.002±0.0006	0.003±0.0007	0.04	0.04	1.23

**Metabolites**	**Average (Control) (%)**	**Average (GBH-exposed) (%)**	**p-value**	**Adjusted p-value**	**Fold Change**
1_4-Lactone	0.01±0.002	0.01±0.004	0.04	0.04	1.21
2,6-DIMETHYLNAPHTHALENE	0.001±0.0004	0.002±0.0006	0.04	0.04	1.40
Fenestrel	0.0006±0.0001	0.0007±0.0002	0.04	0.04	1.26
Piperidine	1.7±0.2	1.8±0.2	0.04	0.04	1.10
Xanthine	0.003±0.2	0.02±0.2	0.04	0.04	0.52

Supplementary Table S3: Metabolites with significant changes in abundance between control and GBH-exposed groups in the gender subgroups.

Metabolites	Average (Control) (%)	Average (GBH-exposed) (%)	p-value	Adjusted p-value	Fold Change
Piperonyl sulfoxide	0.00007±0.00007	0.0006±0.0003	0.004	0.03	8.11
Hydroxyanthraquinone	0.0008±0.0002	0.001±0.0004	0.004	0.03	1.84
butalbital	0.01±0.01	0.0004±0.00009	0.004	0.04	0.03
Amobarbital	0.06±0.07	0.0004±0.0002	0.004	0.04	0.01
Abieticacid	0.0003±0.0002	0.01±0.01	0.004	0.04	36.47
Piceid	0.0005±0.0003	0.005±0.01	0.007	0.03	11.25
GibberellinA14	0.0002±0.00007	0.001±0.001	0.007	0.03	4.32
GibberellinA12	0.0007±0.0004	0.002±0.003	0.007	0.03	3.45
5-Methylcytosine	0.07±0.009	0.1±0.02	0.007	0.03	1.42
5-Ethyl-3,8-dimethyl-1,7-dihydroazulene	0.001±0.0002	0.002±0.0004	0.007	0.03	1.53
4-(2-Hydroxyethyl)phenyl hydrogen sulfate	0.005±0.002	0.001±0.0006	0.007	0.03	0.29
1_7-Dimethylxanthine(paraxanthine)	0.0001±0.00006	0.0003±0.0001	0.007	0.03	2.31
(Z,E)-9,12-Tetradecadienyl acetate	0.0007±0.0003	0.004±0.004	0.007	0.04	5.43
(9S_10S)-10-Hydroxy-9-(phosphonooxy)octadecanoate	0.007±0.003	0.004±0.001	0.007	0.04	0.49
(4R,5S,9S,10R,12S,13S)-1,5,9-Trimethyl-11,14,15,16-tetraoxatetracyclo[10.3.1.0~4,13~.0~8,13~]hexadecan-10-ol	0.0001±0.00004	0.0005±0.0006	0.007	0.04	4.10
Dihydrokavain	0.0001±0.00004	0.0003±0.0001	0.007	0.04	2.57
(2S)-Flavan-4-ol	0.0003±0.0003	0.006±0.01	0.007	0.04	22.93
Rishitin	0.0009±0.0002	0.001±0.0005	0.01	0.02	1.7
Nobiletin	0.0008±0.0006	0.02±0.03	0.01	0.03	22.41
n-Butyl lactate	0.0009±0.00007	0.001±0.0004	0.01	0.03	1.45
L-gamma-Glutamyl-L-leucine	0.009±0.009	0.0001±0.00006	0.01	0.03	0.01
L-(+)-Arginine	0.06±0.07	0.2±0.09	0.01	0.03	3.09
Homoarginine	0.004±0.0003	0.005±0.0006	0.01	0.03	1.27
Falcarindiol	0.002±0.0002	0.003±0.0007	0.01	0.03	1.46
azarole	0.0004±0.00008	0.0006±0.0002	0.01	0.03	1.53
apronalide	0.002±0.0006	0.004±0.002	0.01	0.03	2.05
Androst-4-en-3-one	0.0005±0.0002	0.005±0.007	0.01	0.03	9.82
9-cis-Retinoicacid	0.0050.002	0.030.04	0.01	0.03	6.76
3,4-Dihydroxybenzenesulfonic acid	0.02±0.008	0.007±0.008	0.01	0.03	0.28
1,4-dihydroxy-1,4-dimethyl-7-(propan-2-ylidene)-decahydroazulen-6-one	0.002±0.0003	0.003±0.0008	0.01	0.03	1.42

Metabolites	Average (Control) (%)	Average (GBH-exposed)) (%)	p-value	Adjusted p-value	Fold Change
[STox]3-oxo-13_17-secoandrost-4-ene-17_13alpha-lactone	0.0005±0.0002	0.002±0.002	0.01	0.03	4.74
(3R)-beta-Leucine	0.002±0.0004	0.002±0.0006	0.01	0.04	1.51
Thebaine	0.0002±0.00007	0.0003±0.00007	0.01	0.04	1.79
Epinephrine(OrL-Adrenaline)	0.0006±0.0004	0.0002±0.0001	0.01	0.04	0.29
S-3-oxodecanoyl cysteamine	0.02±0.03	0.0005±0.0003	0.02	0.03	0.03
Valylvaline	0.002±0.002	0.00004±0.00002	0.02	0.03	0.02
Promegestone	0.3±0.06	0.4±0.1	0.02	0.03	1.53
Sulcatone	0.007±0.002	0.01±0.009	0.02	0.03	2.05
Norethindrone	0.0002±0.0001	0.002±0.002	0.02	0.03	6.78
Sebacicacid	0.0005±0.0004	0.00008±0.00002	0.02	0.03	0.16
N-Carbamoylputrescine	0.0003±0.0003	0.0009±0.0004	0.02	0.03	2.70
N_N-Dimethylsphing-4-enine	0.0002±0.00005	0.001±0.002	0.02	0.03	4.73
Naphthalene	0.006±0.002	0.008±0.001	0.02	0.03	1.39
L-N2-(2-Carboxyethyl)arginine	0.004±0.004	0.01±0.002	0.02	0.03	2.51
N1-Methyl-2-pyridone-5-carboxamide	0.08±0.08	0.2±0.1	0.02	0.03	2.39
L-Octanoylcarnitine	0.0001±0.00007	0.0004±0.0002	0.02	0.03	2.63
HMBA	0.08±0.09	0.0009±0.0004	0.02	0.03	0.01
L-2-Amino-3-oxobutanoicacid	0.0004±0.0002	0.0002±0.0001	0.02	0.03	0.50
Glycidyl Stearate	0.006±0.002	0.003±0.0005	0.02	0.03	0.44
Helenalin	0.003±0.0007	0.002±0.0004	0.02	0.03	0.66
Guaiazulene	0.0009±0.0001	0.002±0.0005	0.02	0.03	1.68
Desonide	0.001±0.0007	0.003±0.0009	0.02	0.03	1.99
dehydroretinaldehyde	0.002±0.0007	0.004±0.002	0.02	0.03	1.93
D-Arginine	0.007±0.006	0.02±0.006	0.02	0.03	2.48
CYCLO(-SER-TYR)	0.0007±0.0005	0.00006±0.00002	0.02	0.03	0.08
Brassylic acid	0.006±0.002	0.003±0.0005	0.02	0.03	0.48
Benzylideneacetone	0.006±0.001	0.01±0.003	0.02	0.03	1.52
Androstenedione	0.0002±0.0001	0.0006±0.0005	0.02	0.03	2.60
4-Coumarylalcohol	0.0002±0.00007	0.0004±0.0002	0.02	0.03	2.56
9-Hydroxy-10-undecenoic acid	0.0007±0.0003	0.003±0.003	0.02	0.03	3.85
15-Deoxy-Δ12,14-prostaglandin A1	0.05±0.03	0.4±0.4	0.02	0.03	6.79
5D-5-O-Methyl-2_3_5/4_6-pentahydroxycyclohexanone	0.0007±0.0003	0.0003±0.0001	0.02	0.03	0.46
4-Hydroxy-3-(sulfooxy)benzoic acid	0.005±0.005	0.01±0.004	0.02	0.04	2.54
(2E)-2-Tridecene-4,6,8-triyn-1-ol	0.007±0.001	0.01±0.003	0.02	0.04	1.52
20alpha-Hydroxy-4-pregnen-3-one	0.003±0.001	0.005±0.002	0.02	0.04	1.69
(+)-exo-5-Hydroxycamphor	0.0005±0.0005	0.002±0.001	0.02	0.04	3.00
2'_4'-Dihydroxyacetophenone	0.002±0.0003	0.004±0.003	0.02	0.04	1.77
11-cis-retinal	0.03±0.007	0.06±0.04	0.02	0.04	2.51

Metabolites	Average (Control) (%)	Average (GBH-exposed) (%)	p-value	Adjusted p-value	Fold Change
[SP]Sphinganine-1-phosphate	0.01±0.004	0.006±0.002	0.02	0.04	0.53
[FA(20:4)]17R_18S-epoxy-5Z_8Z_11Z_14Z-eicosatetraenoicacid	0.01±0.003	0.02±0.009	0.02	0.04	1.89
L-gamma-Glutamyl-L-hypoglycin	0.0002±0.0002	0.00002±0.000006	0.02	0.04	0.07
(2S,4S)-4-Amino-2-hydroxy-2-methylpentanedioic acid	0.002±0.0008	0.003±0.001	0.02	0.04	1.77
(-)-bisdechlorogeodin	0.0009±0.0003	0.0004±0.0003	0.02	0.04	0.45
cyclandelate	0.004±0.0006	0.005±0.002	0.02	0.04	1.45
Ubiquinone-1(CoQ1)	0.002±0.002	0.07±0.2	0.03	0.03	42.43
Pregna-4_9(11)-diene-3_20-dione	0.0007±0.0001	0.009±0.02	0.03	0.03	13.17
Leukotriene B4 dimethylamide	0.0007±0.0002	0.001±0.0006	0.03	0.03	1.91
Indole-3-ethanol	0.0005±0.0003	0.002±0.001	0.03	0.03	4.22
D-Proline	0.2±0.2	0.6±0.2	0.03	0.03	3.64
Citric acid	0.4±0.2	0.2±0.2	0.03	0.03	0.48
cis-3-Hexenyl phenylacetate	0.007±0.002	0.02±0.01	0.03	0.04	2.36
CALYCANTHINE	0.0001±0.0001	0.0004±0.0004	0.03	0.04	3.03
Boldione	0.002±0.001	0.007±0.007	0.03	0.04	3.21
androstenol	0.0003±0.0002	0.001±0.001	0.03	0.04	4.82
2-Isobutoxynaphthalene	0.007±0.002	0.01±0.004	0.03	0.04	1.51
2,6-Di-iso-propylnaphthalene	0.002±0.0003	0.003±0.0008	0.03	0.04	1.64
2-(acetylamino)-3-(1H-indol-3-yl)propanoic acid	0.001±0.0005	0.0007±0.0003	0.03	0.04	0.53
1-Carbapen-2-em-3-carboxylicacid	0.01±0.01	0.03±0.02	0.03	0.04	2.90
11-cis-Retinol	0.02±0.005	0.01±0.003	0.03	0.04	0.66
1,5-DAN	0.04±0.02	0.07±0.01	0.03	0.04	1.57
1,1,6-Trimethyl-1,2-dihydronaphthalene	0.001±0.001	0.0002±0.00008	0.03	0.04	0.13
[ST(2:0)]androstan-3beta_17beta-diol	0.0004±0.0003	0.001±0.0009	0.03	0.04	3.20
Agomelatine	0.0004±0.0004	0.0001±0.00009	0.03	0.04	0.24
(2E)-2-(hydroxymethyl)-3-[3-oxo-5-(propan-2-yl)-1,3,4,5,6,7-hexahydro-2-benzofuran-4-yl]prop-2-enoic acid	0.004±0.001	0.002±0.0006	0.03	0.04	0.57
Pinidine	0.0008±0.0008	0.00001±0.000002	0.04	0.04	0.02
(S)-2-hydrazino-3-(4-hydroxy-3-methoxyphenyl)-2-methylpropionic acid	0.00002±0.00001	0.00004±0.00002	0.04	0.04	2.13
(R)-2-Methylimino-1-phenylpropan-1-ol	0.0001±0.00007	0.0006±0.0003	0.04	0.04	5.80

**Metabolites**	**Average (Control) (%)**	**Average (GBH-exposed) (%)**	**p-value**	**Adjusted p-value**	**Fold Change**
3-Oxalomalate*	0.00206±0.0005	0.002±0.0003	0.04	0.04	1.21

*Male rat subgroup

Supplementary Table S4: List of DEMs in the combined rat group validated by LC-PRM-MS including m/z, and fold change (FC) for the cull scan and PRM validation.

Metabolites	m/z	Transition ion fragments	FC (Full scan)	Log2FC (Full scan)	FC (PRM)	Log2FC (PRM)
Xanthine	153.0404	154.0494,153.0655,155.0527	0.52	-0.93	0.47	-1.10
Dihydrokavain	233.1169	215.1789,205.0642,91.0540	1.05	0.08	17.94	4.17
Choline	104.1065	104.1067,105.1101,103.0387	1.20	0.26	1.30	0.38
Paraxanthine	181.0718	181.0715,171.0131,152.9397	1.23	0.30	1.33	0.42
Fenestrel	245.1533	163.1479,83.0854,121.1010	1.26	0.34	1.36	0.44
5-Methylcytosine	126.0658	126.0658,127.0386,83.0602	1.39	0.47	1.10	0.14
235BBF3K97	269.2260	269.2257,157.1009,199.1477	1.43	0.51	1.53	0.61
Falcarindiol	261.1846	91.0540,121.1009,244.1323	1.44	0.52	1.17	0.22
Promegestone	327.2315	171.1164,185.1320,213.1632	1.47	0.56	1.19	0.26
Rishitin	223.1688	167.0852,195.0800,223.1476	1.48	0.56	1.89	0.92
[FA(20:4)]17R_18S-epoxy-5Z_8Z_11Z_14Z-eicosatetraenoicacid	319.2263	81.0698,318.2139,139.1114	1.52	0.60	1.04	0.06
VIRILON	413.3051	57.0700,67.0542,367.2984	1.52	0.61	1.24	0.31
R.g.-Keto III	629.4563	325.1884,119.0853,335.1884	1.54	0.62	1.41	0.50
D-Glutamate	148.0601	84.0442,130.0496,102.0547	1.58	0.66	2.39	1.26
Glycerol	93.0543	62.9900, 72.9370, 91.0540	1.61	0.69	1.65	0.72
12-oxo Phytodienoic Acid	293.2109	81.0697,95.0853,262.1253	1.62	0.70	1.34	0.42
oxeladin	336.2530	308.8992,271.2049,95.0853	1.63	0.70	3.22	1.69
Diethyl phthalate	223.0963	149.0230,73.0646,172.1116	1.70	0.76	2.41	1.27
dehydroretinaldehyde	283.2055	252.1143,283.2051,176.1429	1.71	0.78	1.25	0.32
cis-3-Hexenyl phenylacetate	219.1376	219.1740,91.0541,119.0853	1.96	0.97	1.19	0.25

**Metabolites**	**m/z**	**Transition ion fragments**	**FC (Full scan)**	**Log2FC (Full scan)**	**FC (PRM)**	**Log2FC (PRM)**
(+/-)-6-Hydroxy-3-oxo-alpha-ionone	223.1326	223.1476,167.0852,195.0800	2.00	1.00	4.66	2.22
Auraptene	299.1643	283.2050,171.1163,131.0852	2.07	1.05	5.21	2.38
D-Arginine	175.1186	177.0605,175.1185,70.065	2.10	1.07	3.07	1.62
3alpha_11beta-Dihydroxy-5alpha-androstane-17-one	307.2267	81.0697,67.0542,95.0853	2.12	1.08	4.43	2.15
L-N2-(2-Carboxyethyl)arginine	247.1399	175.1185,188.0912,247.1395	2.24	1.16	2.58	1.37
Forskolin	411.2355	154.1219,63.8566,251.1407	2.36	1.24	4.14	2.05
[FA(20:4)]5Z_8Z_11Z_14Z-eicosatetraenoicacid	305.2468	221.1531,121.1009,93.0697	2.82	1.50	1.22	0.29
4-Hydroxy-3-(sulfooxy)benzoic acid	234.9896	151.9762,179.9710,133.9657	2.87	1.52	6.22	2.64
L-(+)-Arginine	175.1186	177.0605,175.1185,70.065	3.03	1.61	3.07	1.62
19-Oxoandrost-4-ene-3_17-dione	301.1796	283.2053,255.2105,117.0696	3.35	1.75	2.40	1.26
N_N-Dimethylsphing-4-enine	328.3209	291.2313,96.9949,69.0698	3.62	1.85	1.81	0.85
sulbenox	211.0534	211.0530,198.9713,203.9379	3.98	1.99	3.85	1.94
DAPSONE HYDROXYLAMINE	265.0639	232.1331,207.1011,139.1008	4.81	2.26	5.14	2.36
Diethanolamine	106.0859	104.1068,105.0031,60.0809	5.33	2.42	52.05	5.70
Androst-4-en-3-one	273.2209	138.5293,165.1270,273.2206	6.57	2.72	5.75	2.52
(2S)-Flavan-4-ol	227.1063	121.1009,149.0119,203.9380	18.45	4.21	8.82	3.14
Ubiquinone-1(CoQ1)	251.1274	205.1219,250.1770,233.1166	22.09	4.47	42.91	5.42

Supplementary Table S5: List of DEMs in the female group validated by LC-PRM-MS including m/z, transition fragment ions, and fold change (FC) for the full scan and PRM validation.

Metabolites	m/z	Transition ion fragments	FC (Full scan)	Log2FC (Full scan)	FC (PRM)	Log2FC (PRM)
S-3-oxodecanoyl cysteamine	246.1520	244.2629,226.2523,58.0653	0.03	0.04	-5.17	-4.79
butalbital	225.1229	148.0213,224.0371,143.0012	0.03	0.05	-4.86	-4.21
CYCLO(-SER-TYR)	251.1023	193.0835,174.0545,94.0650	0.08	0.35	-3.69	-1.53
2-(acetylamino)-3-(1H-indol-3-yl)propanoic acid	247.1075	247.0859,159.0913,188.0701	0.53	0.54	-0.92	-0.88
(-)-bisdechlorogeodin	331.0812	119.0853,331.0804,159.1164	0.45	0.67	-1.15	-0.59
[SP]Sphinganine-1-phosphate	382.2715	265.2713,82.0649,365.1780	0.53	0.69	-0.93	-0.53
L-gamma-Glutamyl-L-leucine	261.1442	187.1476,244.1323,91.0540	0.01	0.71	-6.12	-0.49
Epinephrine(OrL-Adrenaline)	184.0966	159.9907,155.0699,147.9740	0.29	0.78	-1.76	-0.36
[FA(20:4)]17R_18S-epoxy-5Z_8Z_11Z_14Z-eicosatetraenoicacid	319.2263	81.0698,318.2139,139.1114	1.89	1.03	0.92	0.04
dehydroretinaldehyde	283.2055	252.1143,283.2051,176.1429	1.93	1.09	0.95	0.13
Promegestone	327.2315	171.1164,185.1320,213.1632	1.53	1.12	0.62	0.16
11-cis-retinal	285.2210	285.2205,267.2100,183.1164	2.51	1.15	1.33	0.20
Norethindrone	299.2002	299.1998,253.1944,131.0852	6.78	1.18	2.76	0.24
Falcarindiol	261.1846	91.0540,121.1009,244.1323	1.46	1.20	0.55	0.26
Guaiazulene	199.1477	60.0445,198.1848,106.9635	1.68	1.25	0.75	0.32
D-Proline	116.0701	70.0650,116.0704,118.0860	3.64	1.26	1.86	0.34
cis-3-Hexenyl phenylacetate	219.1376	219.1740,91.0541,119.0853	2.36	1.31	1.24	0.39
Boldione	285.1847	285.2205,267.2100,183.1164	3.21	1.45	1.68	0.54
Paraxanthine	181.0718	181.0715,171.0131,152.9397	2.31	1.60	1.21	0.68
Homoarginine	189.1342	173.0294,145.0349,130.0860	1.27	1.61	0.35	0.69
2'_4'-Dihydroxyacetophenone	153.0543	153.0542,126.0625,112.0471	1.77	1.62	0.83	0.70
(S)-2-hydrazino-3-(4-hydroxy-3-methoxyphenyl)-2-methylpropionic acid	241.1181	107.0700,133.0857,89.0596	2.13	1.62	1.09	0.70
(2S,4S)-4-Amino-2-hydroxy-2-methylpentanedioic acid	178.0707	114.0547,132.0652,96.0442	1.77	1.62	0.83	0.70
N_N-Dimethylsphing-4-enine	328.3209	291.2313,96.9949,69.0698	4.73	1.68	2.24	0.75
GibberellinA14	349.2008	331.2260,133.1009,271.2054	4.32	1.81	2.11	0.86

**Metabolites**	**m/z**	**Transition ion fragments**	**FC (Full scan)**	**Log2FC (Full scan)**	**FC (PRM)**	**Log2FC (PRM)**
GibberellinA12	333.2059	189.1269,253.1944,299.1998	3.45	1.87	1.79	0.90
Hydroxyanthraquinone	225.0543	225.1482,207.1375,189.1633	1.84	2.15	0.88	1.10
N1-Methyl-2-pyridone-5-carboxamide	153.0655	80.9448,100.8959,100.8959	2.39	2.30	1.26	1.20
Rishitin	223.1688	167.0852,195.0800,223.1476	1.70	2.50	0.77	1.32
1-Carbapen-2-em-3-carboxylicacid	154.0495	154.0494,72.9369,120.9607	2.90	2.62	1.54	1.39
azarole	237.1121	207.1373,195.1374,181.0855	1.53	3.01	0.61	1.59
L-N2-(2-Carboxyethyl)arginine	247.1399	175.1185,188.0912,247.1395	2.51	3.07	1.33	1.62
cyclandelate	277.1798	223.0748,277.1768,81.0697	1.45	3.18	0.54	1.67
(R)-2-Methylimino-1-phenylpropan-1-ol	164.1068	163.0750,136.9580,141.9559	5.80	3.59	2.54	1.84
D-Arginine	175.1186	177.0605,175.1185,70.065	2.48	3.74	1.31	1.90
L-(+)-Arginine	175.1186	177.0605,175.1185,70.065	3.09	3.74	1.63	1.90
Androstenedione	287.2004	118.1223,83.0853,270.2421	2.60	4.27	1.38	2.09
4-Hydroxy-3-(sulfooxy)benzoic acid	234.9896	151.9762,179.9710,133.9657	2.54	5.87	1.34	2.55
Thebaine	312.1593	296.2213,91.0540,159.1162	1.79	8.42	0.84	3.07
Piperonyl sulfoxide	325.1830	179.0851,223.0591,325.2164	8.11	9.07	3.02	3.18
Androst-4-en-3-one	273.2209	138.5293,165.1270,273.2206	9.82	9.68	3.30	3.28
androstenol	275.2368	274.2734,104.1068,149.5484	4.82	11.61	2.27	3.54
(2S)-Flavan-4-ol	227.1063	121.1009,149.0119,203.9380	22.93	14.12	4.52	3.82
Nobiletin	403.1383	375.0258,188.0703,259.1683	22.41	21.69	4.49	4.44
Dihydrokavain	233.1169	215.1789,205.0642,91.0540	2.57	23.70	1.36	4.57
Ubiquinone-1(CoQ1)	251.1274	205.1219,250.1770,233.1166	42.43	72.64	5.41	6.18

Supplementary Table S6: List of DEMs shown to be implicated in some important diseases and functions based on IPA results**.**

**Combined group**			
**Metabolites**	**p-values [1]**	**Fold Change**	**AUC Value**
Choline	0.01	1.20	0.80
D-Arginine	0.02	2.10	0.77
L-(+)-Arginine	0.02	3.03	0.83
Glycerol	0.02	1.61	0.79
Paraxanthine	0.03	1.23	0.76
Xanthine	0.04	0.52	0.75
**Female subgroup**			
Paraxanthine	0.007	2.31	0.95
L-(+)-Arginine	0.01	3.09	0.93
Epinephrine	0.01	0.29	0.93
D-Arginine	0.02	2.48	0.91
Androstenedione	0.02	2.60	0.88

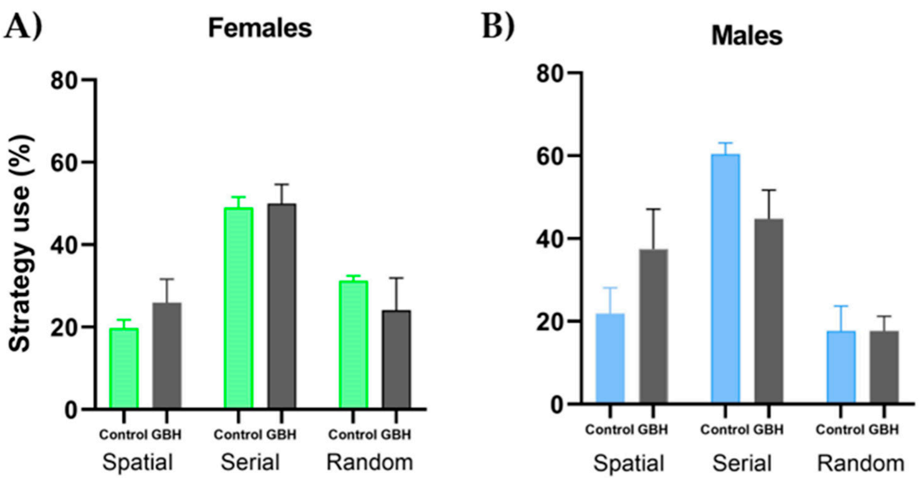

Supplementary Figure S1. Effect of chronic exposure to GBH on search strategy of A) females and B) males studied on Barnes maze (n = 6 - 7 rats/group). Results are presented as mean ± SEM.

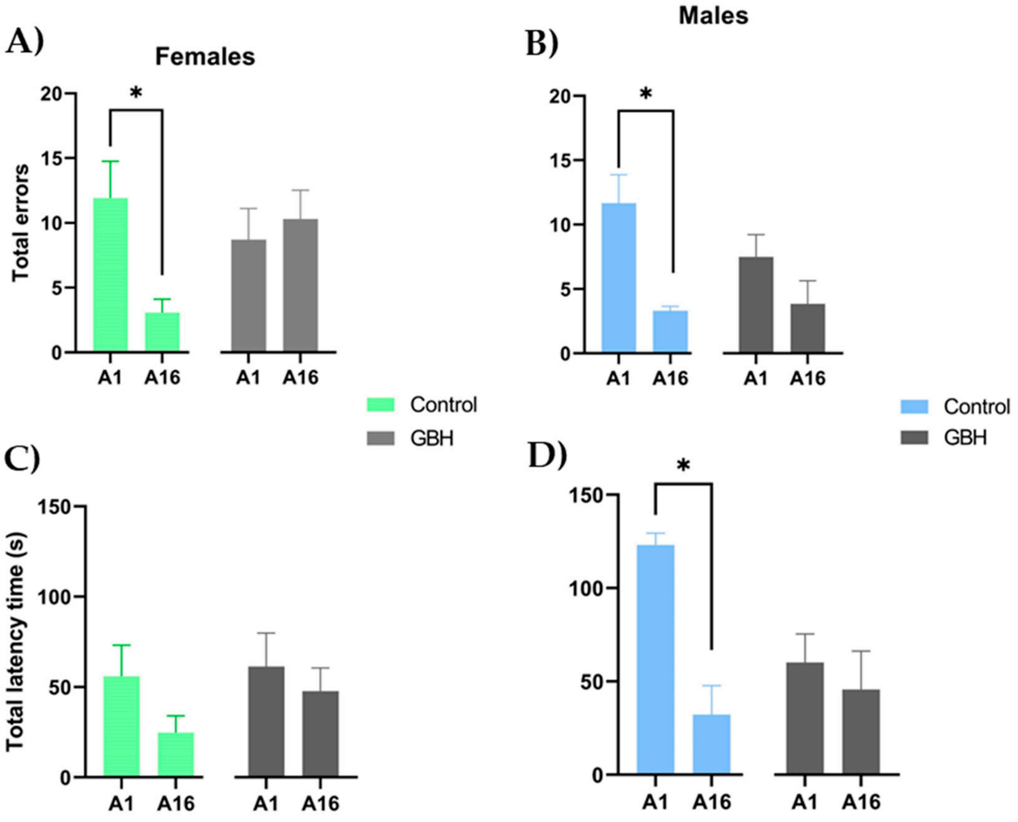

Supplementary Figure S2. Effect of chronic exposure to GBH on total errors A and B) and latency C and D) to solve the Barnes maze in rats (n = 6 - 7 rats/group). Results are presented as mean ± SEM. *$p < 0.05$.

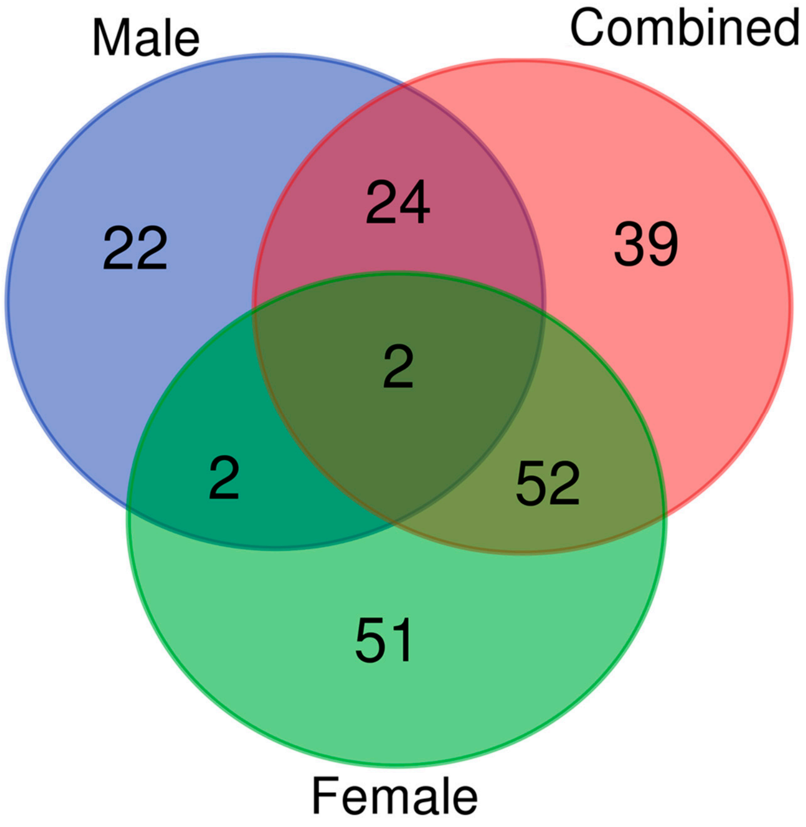

Supplementary Figure S3. Venn diagram shows overlapping and unique significant metabolites in the whole data set and gender comparison between control and GBH-exposed rats. The orange circle represents the significant metabolites in the combined comparison, while the blue and green circles represent the significant metabolites in the male and female comparisons respectively. The result shows 2 common metabolites in all comparisons, with 39, 22 and 51 unique significant metabolites in the combined, male and female comparisons respectively.

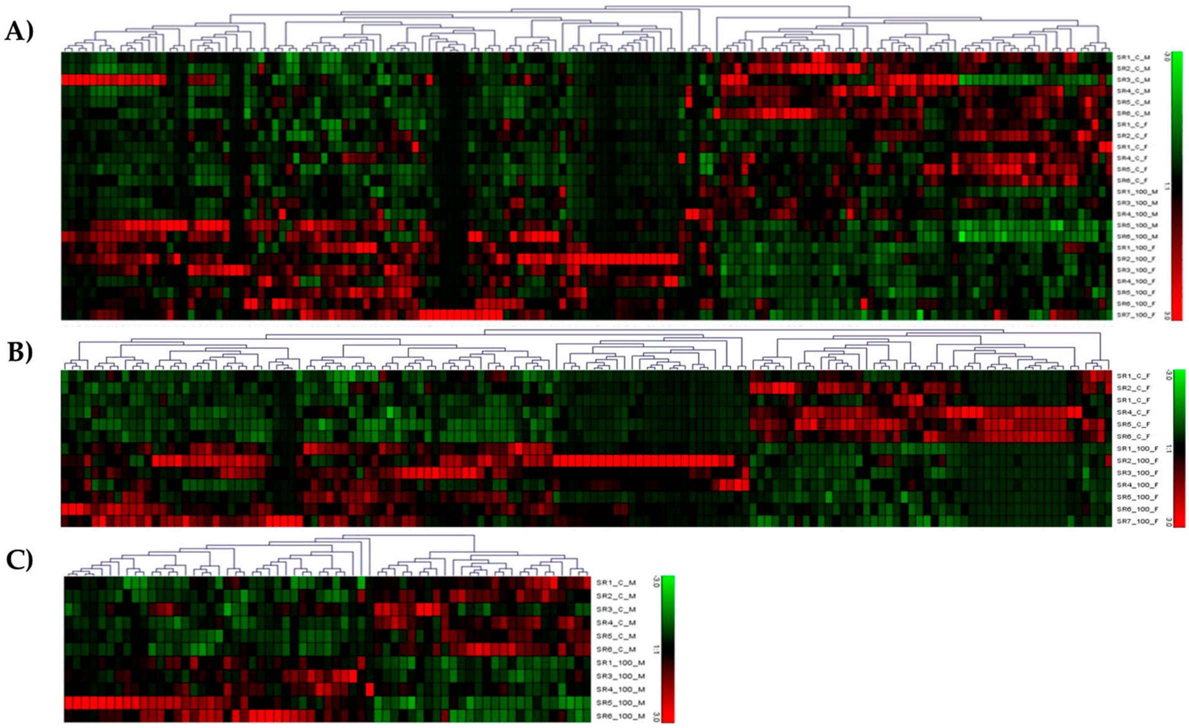

Supplementary Figure S4. Heatmap of significant metabolites in **A)** control and GBH-exposed groups in the combined sample group, **B)** female rat samples, and **C)** male rat samples (C_M = Control (male); C_F = Control (female); 100_M = GBH-exposed (male); 100_F = GBH-exposed (female)). The red color indicates up regulated while the green color indicates down regulated metabolites.

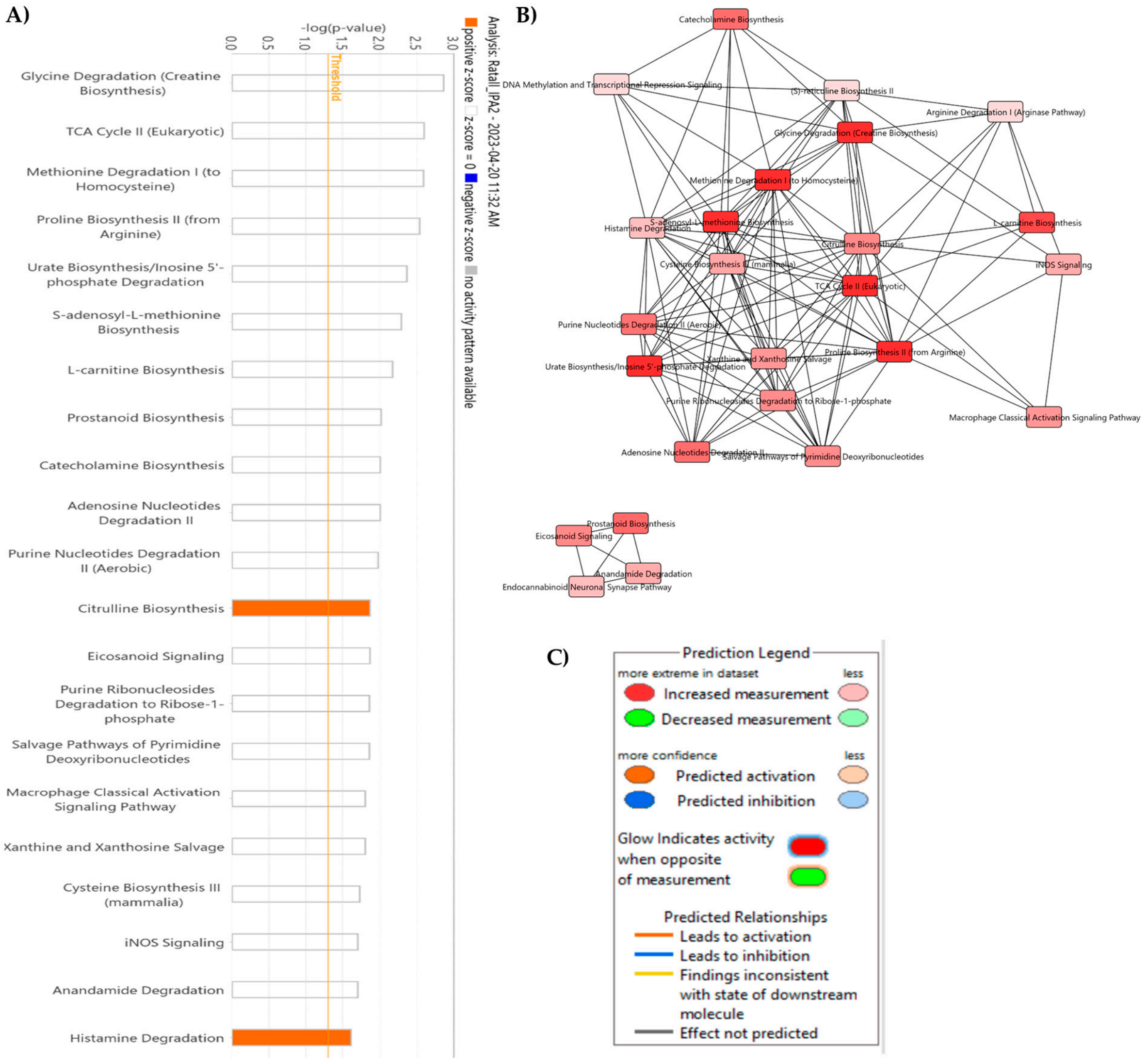

Supplementary Figure S5. A) **Top** Canonical pathways, B) Pathways network, C) Prediction legends. (Red indicates increased measurement, Green represents decreased measurement, Blue represents predicted inhibition, and Orange represents predicted activation).

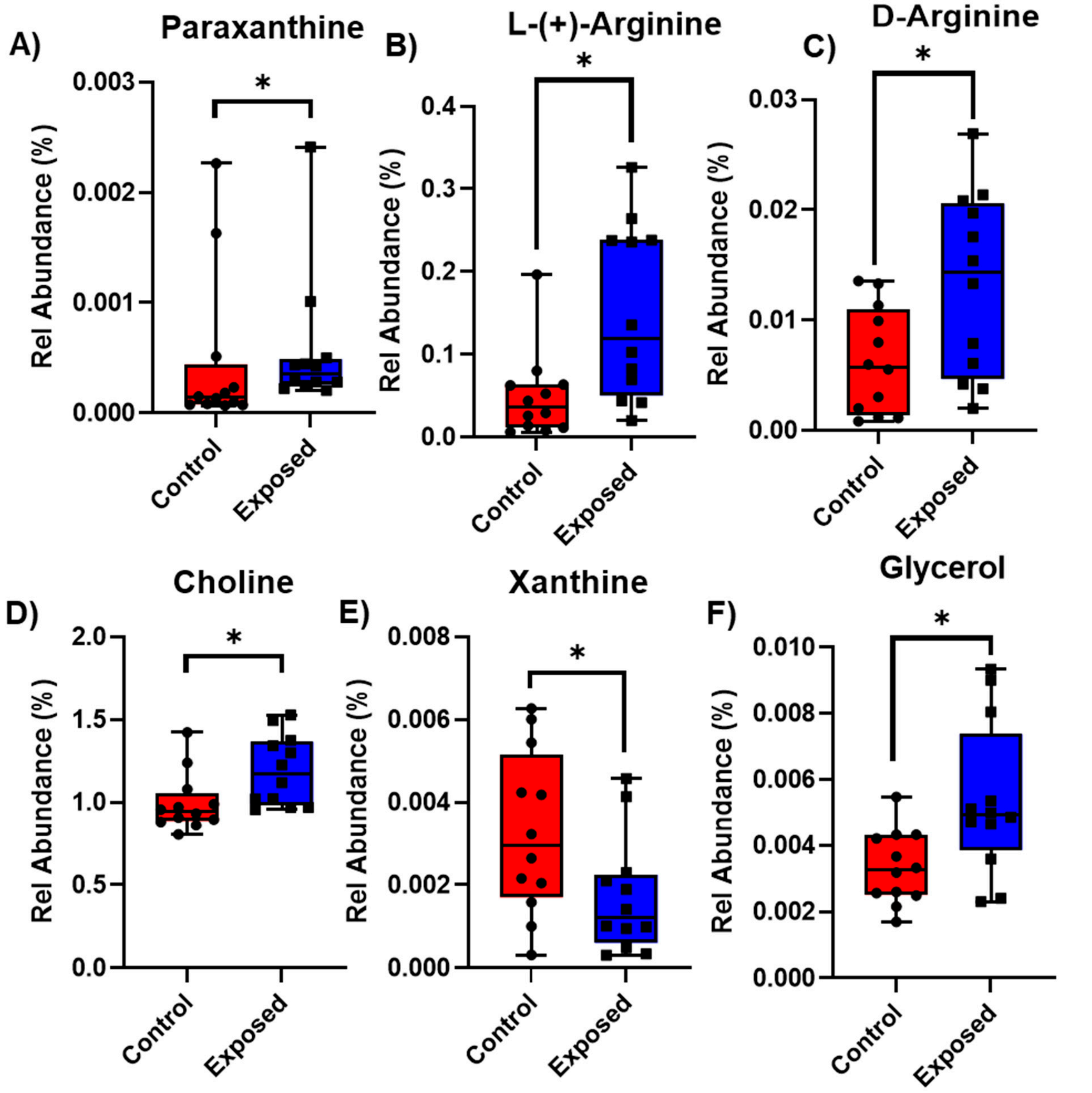

Supplementary Figure S6: Boxplot showing the changes in the DEMs from combined group comparison between control to GBH-exposed. Five metabolites were up regulated, and one metabolite was down regulated. A) Paraxanthine (Upregulated), B) L-(+)-Arginine (Upregulated), C) D-Arginine (Upregulated), D) Choline (Upregulated), E) Xanthine (Downregulated), F) Glycerol (Upregulated).

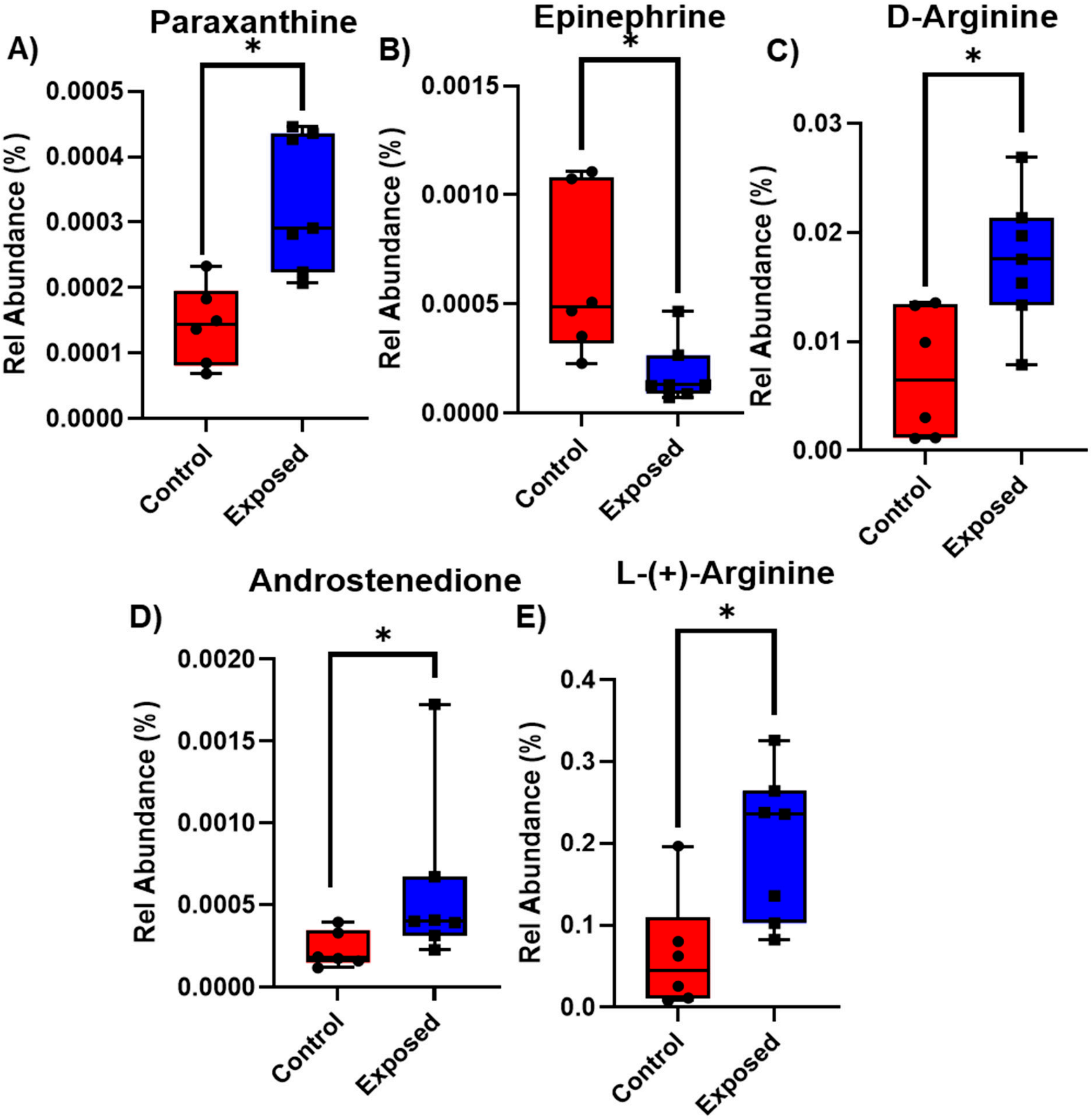

Supplementary Figure S7. Boxplot showing the changes in the control and GBH-exposed DEMs in female rats. Four metabolites were up regulated, and one metabolite was down regulated. A) Paraxanthine (Upregulated), B) Epinephrine (Downregulated), C) D-Arginine (Upregulated), D) Androstenedione (Upregulated), E) L-(+)-Arginine (Upregulated).
